# Germline mutations of breast cancer susceptibility genes through expanded genetic analysis in unselected Colombian patients

**DOI:** 10.1186/s40246-024-00623-7

**Published:** 2024-06-18

**Authors:** Diana Carolina Sierra-Díaz, Adrien Morel, Dora Janeth Fonseca-Mendoza, Nora Contreras Bravo, Nicolas Molano-Gonzalez, Mariana Borras, Isabel Munevar, Mauricio Lema, Henry Idrobo, Daniela Trujillo, Norma Serrano, Ana Isabel Orduz, Diego Lopera, Jaime González, Gustavo Rojas, Paula Londono-De Los Ríos, Ray Manneh, Rodrigo Cabrera, Wilson Rubiano, Jairo de la Peña, María Catalina Quintero, William Mantilla, Carlos M. Restrepo

**Affiliations:** 1 School of Medicine and Health Sciences, Center for Research in Genetics and Genomics (CIGGUR), Institute of Translational Medicine (IMT), Universidad Del Rosario, Bogotá, Colombia; 2 Fundación Cardioinfantil, Instituto de Cardiología, Bogotá, Colombia; 3 Clínica de Oncología Astorga, Medellín, Colombia; 4 Centro Médico Julián Coronel, Cali, Colombia; 5 Hospital Internacional de Colombia HIC, Piedecuesta, Colombia; 6 Oncólogos del Occidente S.A.S, Manizales, Colombia; 7 SOHEC, Sociedad de Oncología y Hematología del Cesar, Valledupar, Colombia; 8 Hospital Universitario Mayor Méderi, Bogotá, Colombia; 9 Clinical Research Group, School of Medicine and Health Science, Universidad del Rosario, Bogotá, Colombia; 10 Fundación CTIC-Fundación Cardioinfantil, Instituto de Cardiología, Bogotá, Colombia; 11 Laboratorio de Biología Molecular y Pruebas Diagnósticas de Alta Complejidad, Fundación Cardioinfantil-Instituto de Cardiología, Bogotá, Colombia; 12 Integrative IPS, Bogotá, Colombia; 13 Oncologos del Occidente SAS, Pereira, Colombia

**Keywords:** Unselected breast cancer, Whole exome sequencing, Pathogenic germline variants, Minigene assay

## Abstract

**Background:**

In Colombia and worldwide, breast cancer (BC) is the most frequently diagnosed neoplasia and the leading cause of death from cancer among women. Studies predominantly involve hereditary and familial cases, demonstrating a gap in the literature regarding the identification of germline mutations in unselected patients from Latin-America. Identification of pathogenic/likely pathogenic (P/LP) variants is important for shaping national genetic analysis policies, genetic counseling, and early detection strategies. The present study included 400 women with unselected breast cancer (BC), in whom we analyzed ten genes, using Whole Exome Sequencing (WES), know to confer risk for BC, with the aim of determining the genomic profile of previously unreported P/LP variants in the affected population. Additionally, Multiplex Ligation-dependent Probe Amplification (MLPA) was performed to identify Large Genomic Rearrangements (LGRs) in the *BRCA1/2* genes. To ascertain the functional impact of a recurrent intronic variant (*ATM* c.5496 + 2_5496 + 5delTAAG), a minigene assay was conducted.

**Results:**

We ascertained the frequency of P/LP germline variants in *BRCA2* (2.5%)*, ATM* (1.25%)*, BRCA1* (0.75%), *PALB2* (0.50%), *CHEK2* (0.50%), *BARD1* (0.25%)*,* and *RAD51D* (0.25%) genes in the population of study. P/LP variants account for 6% of the total population analyzed. No LGRs were detected in our study. We identified 1.75% of recurrent variants in *BRCA2* and *ATM* genes. One of them corresponds to the *ATM* c.5496 + 2_5496 + 5delTAAG. Functional validation of this variant demonstrated a splicing alteration probably modifying the Pincer domain and subsequent protein structure.

**Conclusion:**

This study described for the first time the genomic profile of ten risk genes in Colombian women with unselected BC. Our findings underscore the significance of population-based research, advocating the consideration of molecular testing in all women with cancer.

**Supplementary Information:**

The online version contains supplementary material available at 10.1186/s40246-024-00623-7.

## Background

Breast cancer (BC) is the most frequently diagnosed malignant neoplasm and the leading cause of death from cancer in women [1].

BC incidence in Latin-American (LATAM) countries is generally lower when compared to high-income countries (HIC). However, unlike in HIC, BC-related mortality has not shown a declining trend in LATAM and has, in fact, increased in some countries over the past decade (https://gco.iarc.fr/). In Colombia, the observed 5-year survival rate for BC was 72% according to the CONCORD3 trial [2], highlighting a significant disparity between LATAM and HIC. This disparity can be attributed to various factors, including disease characteristics, healthcare system issues, and the availability of early diagnosis programs, among others.

An essential factor in risk assessment and early diagnosis is the recognition of hereditary BC risk, which may account for as much as 10% of all BC cases [3].

Germline cancer risk study can have various approaches, among them, the study of selected populations based on pedigrees with hereditary and familial cancer segregation analysis to identify genes linked with specific risks, or the study of unselected cases. In the latter approach, unselected cases, which involve individuals without consideration of family history or age at diagnosis, enable the calculation of estimates related to germline mutation prevalence, assessment of cancer risk genes, and identification of at-risk relatives, free from ascertainment bias [4].

Worldwide Breast Cancer Association Consortium (BCAC) reported *BRCA1*, *BRCA2*, *ATM*, *PALB2*, *CHEK2, BARD1, RAD51C, RAD51D, CDH1,* and *TP53*, as the main genes for the prediction of hereditary BC risk [5]. Furthermore, BCAC and Cancer Risks Estimates Related to Susceptibility Consortium (CARRIERS), suggested *BRCA1*, *BRCA2*, *ATM*, *PALB2,* and *CHEK2* genes as highly penetrant; both consortiums pinpointed that 10% of BC patients have cancer susceptibility germline mutation [3].

The mutational spectrum of some of these genes has been assessed in Latin American populations in studies of unselected BC, indicating carrier frequencies ranging from 10.7% in Argentina to 25.2% in Brazil [6, 7]. These differences may reflect the high ethnic variability attributed to Latin American populations.

Colombia has a mixed population composed of Amerindian (descendants of indigenous people), European immigrants (mostly Spanish), and Africans, with recent waves of settlements that have included individuals from the Middle East, Romanies, Germans (around World War I and II), and Asian populations [8], although these represent a small minority.

To date, no study in Colombia has comprehensively examined all the genes considered highly significant by the BCAC and CARRIERS consortia. Additionally, there is a lack of data on the mutational spectrum of these genes within the unselected Colombian population. Our research aims to describe for the first time in our country the genomic profile of ten genes risk for breast cancer, in 400 unselected Colombian women with BC, using whole exome sequencing (WES). This population is particularly noteworthy since most studies in our and other Latin American countries have primarily focused on hereditary cases. Our findings uncovered both new and recurrent pathogenic variants. Furthermore, through functional validation, we propose molecular mechanisms that are linked to the etiology of the disease.

## Methods

### Patients

From March 2019 to May 2022 women with BC were included in the trial, in cancer centers located throughout Colombia. The study included women with a diagnosis of invasive BC (within one year from diagnosis) supported with biopsy and immunohistochemical test. Women or their relatives with known *BRCA1* or *BRCA2* mutations were excluded. Patients older than 18 years were invited to participate in this study and those who accepted signed an informed consent.

This study was performed in compliance with the Helsinki Declaration and all experimental procedures were approved by Fundación Cardioinfantil–Instituto de Cardiología and Universidad del Rosario Ethics Committee (approval numbers: 402018 7–11-2018, DVO005 1805-CV1469 3–12-2021, Pfizer: WI241988 – Investigator initiate research, independent review board: 28–08-2018, GF1147 2018).

### Clinical data collection

The clinical and sociodemographic variables collected have been described in supplementary methods.

### Genomic DNA extraction

The quality and quantity of the DNA were evaluated through the measurement of absorbance with a Nanodrop (OD260/280 and OD260/230).

### MLPA (*multiplex ligation-dependent probe amplification*—MLPA)

MLPA was performed using the commercial kit SALSA MLPA Probemix P002-D1 for *BRCA1* and P090-C1 for *BRCA2* (MRC-Holland, Amsterdam). Experimental details have been included as supplementary methods.

### Next generation sequencing (NGS–WES)

Genomic DNA was extracted from peripheral blood samples according to the protocol of the Quick-DNA Miniprep plus kit (Zymo Research, Orange, California, USA). Experimental details of library preparation, WES, bioinformatic analysis and germline variant classification have been included as supplementary methods.

### Segregation analysis

All families with an index case carrier of a pathogenic or likely pathogenic germline variant classified according to the ACMG/AMP, ClinGen, or ENIGMA criteria and confirmed by Sanger sequencing were invited to participate in a segregation analysis and all relatives of the index case (with or without cancer at any age), who were willing to participate in the study, were tested. A total of 36 relatives were included in the family segregation analysis.

### Functional validation of the recurrent intronic variant in *ATM* gene (minigene assay)

Experimental details of minigene assay have been included as supplementary methods.

### Statistical analysis

Qualitative variables are summarized as frequencies and percentages while quantitative variables as medians and interquartile ranges were reported. To assess possible associations with mutation status Kruskal–Wallis test for quantitative variables and the Chi-square independence test for qualitative were used. All statistical analyses were done in software R version 4.3.0 [9].

## Results

### Population of study

We enrolled 400 patients in the study, the median age of diagnosis was 53 years, 55.5% of them were post-menopausal and 60.3% were overweight or obese. The main histologic diagnosis was ductal carcinoma (85.5%), the prevalence of triple-negative BC (TNBC) was 11.5%, the prevalence of metastatic disease was 4%, and 61.1% of the patients met NCCN criteria for hereditary BC testing. Table 1 summarizes the main data obtained from the 400 women with unselected BC.

**Table 1:** Baseline characteristics of the patients

Variable		*n* = 400	%
Age at diagnosis (median–range)		53	43–64
Tumor size (median–range)		20	12–30
Positive nodes (median–range)		0	0–1
Ki67 (median–range)		25	13–45
*Histologic diagnosis*	*Ductal*	342	85,50
	*Lobular*	21	5,25
	*Other*	35	8,75
	*ND*	2	0,50
*ER status*	*Negative*	87	21,75
	*Positive*	308	77
	*ND*	5	1,25
*PR status*	*Negative*	117	29,25
	*Positive*	278	69,5
	*ND*	5	1,25
*HER-2 status*	*Negative*	298	74,5
	*Positive*	95	23,75
	*ND*	7	1,75
*TNBC status*	*No*	349	87,25
	*Yes*	46	11,50
	*ND*	5	1,25
*Nodal stage*	*0*	204	51
	*1*	134	33,50
	*2*	39	9,75
	*3*	14	3,50
	*ND*	9	2,25
*Tumoral stage*	*I*	91	22,75
	*II*	183	45,75
	*III*	104	26
	*IV*	16	4
	*ND*	6	1,50
*Age of menarche (median–range)*		13	12–14
*Born children (median–range)*		2	1,50–3
*Age first born child (median–range)*		23	19–28
*Lactation*	*No*	76	19
	*Yes*	322	80,50
	*ND*	2	0,50
*Menopause*	*No*	173	43,25
	*Yes*	222	55,50
	*ND*	5	1,25
*Age of menopause (median–range)*		50	46–52
*Weight (median–range)*		65	58–73,30
*Height (median–range)*		1,58	1,55–1,63
*BMI (median–range)*		25,96	23,57–29,14
*Overweight–obesity*	*No*	153	39
	*Yes*	241	60,25
	*ND*	3	0,75
*Hormonal contraception exposure*	*No*	201	50,25
	*Yes*	196	49
	*ND*	3	0,75
*HRT*	*No*	372	93
	*Yes*	24	6
	*ND*	4	1
*Current or past smoking*	*No*	296	74
	*Yes*	102	25,50
	*ND*	2	0,50
*Alcohol consumption*	*No*	347	86,75
	*Yes*	50	12,50
	*ND*	3	0,75
*Radiation exposure*	*No*	346	86,50
	*Yes*	25	6,25
	*ND*	29	7,25
*Personal history of cancer*	*No*	377	94,25
	*Yes*	21	5,25
	*ND*	2	0,50
*Family history of cancer*	*No*	120	30
	*Yes*	275	68,75
	*ND*	5	1,25
*fulfill of NCCN criteria v1.2023*	*No*	155	38,75
	*Yes*	243	60,75
	*ND*	2	0,50
*Inheritance*	*Sporadic*	158	39,50
	*Familial*	187	46,75
	*Hereditary*	50	12,50
	*ND*	5	1,25
*P/LP variant*	*No*	376	94
	*Yes*	24	6
*Mutation status*	*No mut*	376	94
	*BRCA*	13	3,25
	*noBRCA*	11	2,75

Tumor size was measured in millimeters (mm); weight is given in kilograms (kg); height is given in meters (m); BMI: body mass index (kg/m^2^); other (mixed, medullary, mucinous, metaplastic, tubular, micropapillary, papillary, adenocarcinoma, apocrine, and cribriform); HRT: hormonal replacement therapy; NCCN criteria v1.2023 [10]; hereditary breast cancer: autosomal dominant inheritance pattern involving at least three generations, consider cancers associated with Lynch syndrome [11]; familial breast cancer: breast cancer with a family history of one or more first- or second-degree relatives with breast cancer that does not fit the hereditary breast cancer definition [11]; P/LP variant: presence of pathogenic or likely pathogenic germline variant; no_mut: absence of P/LP variant; BRCA: P/LP variant identified in genes *BRCA1* or *BRCA2*; no*BRCA*: P/LP variant identified in genes *ATM, BARD1, CHEK2, PALB2* or *RAD51D*

### Germline mutations identified in women with unselected BC

**Fig. 1:**
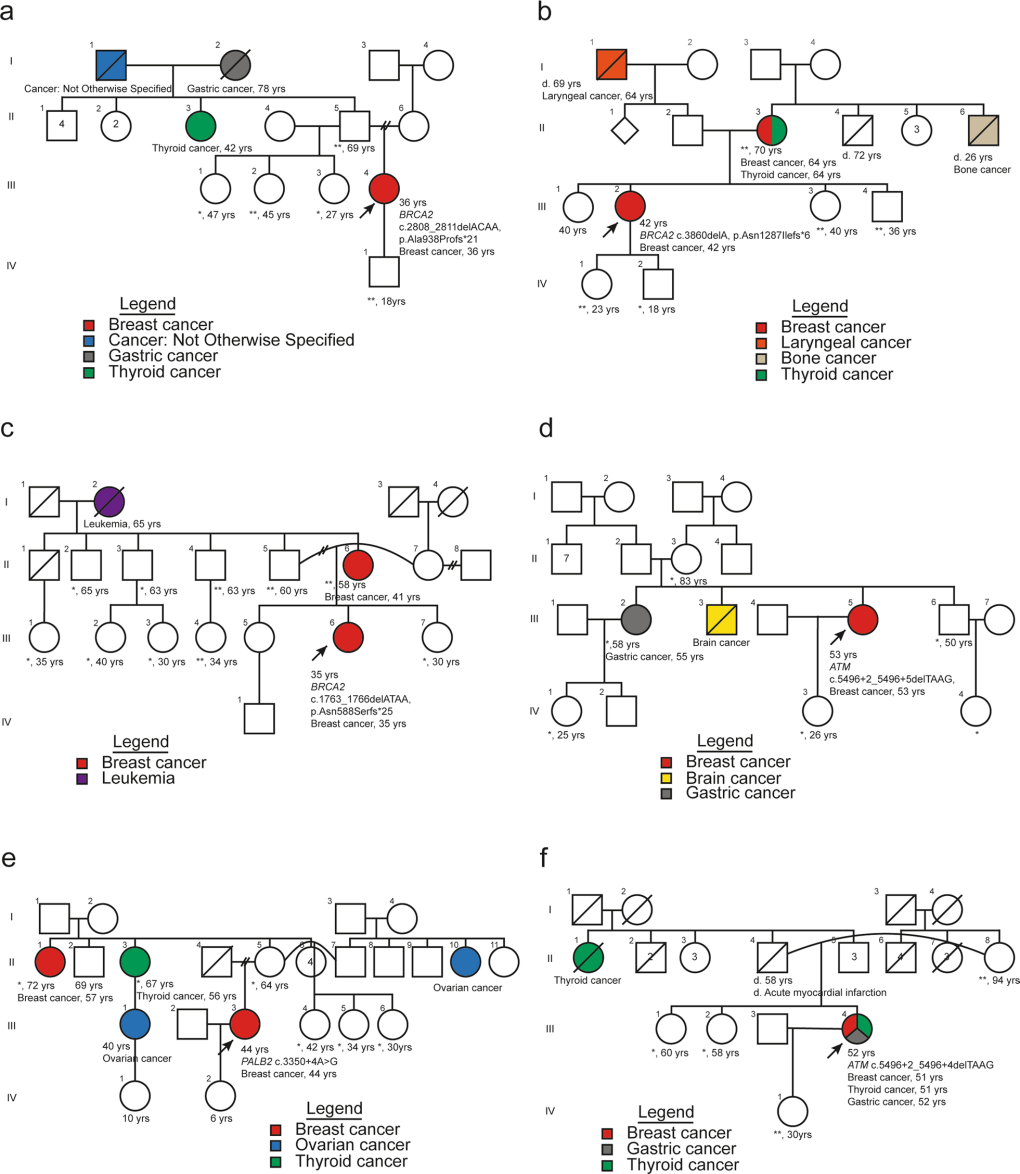
Pedigrees of index cases and their relatives assessed in the segregation analysis. **a** Germline mutation located in *BRCA2* gene: c.2808_2811delACAA, p.Ala938Profs*21; **b** Germline mutation located in *BRCA2* gene: c.3860delA, p.Asn1287Ilefs*6; **c** Germline mutation located in *BRCA2* gene: c.1763_1766delATAA, p.Asn588Serfs*25; **d** and **f** Germline mutation located in *ATM* gene: c.5496 + 2_5496 + 5delTAAG; **e** Germline mutation located in *PALB2* gene: c.3350 + 4A > G; **individuals tested harboring germline mutation; * individuals tested not harboring germline mutation

All 400 women with unselected BC were assessed with ten known cancer genes as follows: *BRCA1*, *BRCA2*, *ATM*, *PALB2*, *CHEK2, BARD1, RAD51C, RAD51D, CDH1,* and *TP53* which were sequenced by WES. 24 (6%) patients had pathogenic or like pathogenic variants (P/LP variants) identified. 18 germline pathogenic variants were identified in 19 individuals (11 in *BRCA1/2* genes and seven in *ATM, BARD1, CHEK2, PALB2*, and *RAD51D* genes). From these variants, 12 were frameshift (67%), four nonsense (22%), and two missense (11%). *PALB2* gene showed two molecular changes that were not reported in ClinVar nor dbSNP database, designated as novel. Three likely pathogenic variants were identified in five women in *ATM, CHEK2,* and *PALB2* genes. Likely pathogenic variants were represented by one missense (33%) and two intronic (67%). All the P/LP variants were in a heterozygous state. The variants are summarized in Tables 2, 3, and supplementary methods Fig. 1.

**Table 2:** Frequency of P/LP variants in the population of study and their distribution by genes

*Genes*	Population of study (*n* = 400). Frequency of patients with mutation (%)	P/LP variants (*n* = 21). Frequency of P/LP variants (%)
*BRCA2*	10 (2.50%)	8 (38.10%)
*ATM*	5 (1.25%)	3 (14.28%)
*BRCA1*	3 (0.75%)	3 (14.28%)
*PALB2*	2 (0.50%)	3 (14.28%)
*CHEK2*	2 (0.50%)	2 (9.52%)
*BARD1*	1 (0.25%)	1 (4.76%)
*RAD51D*	1 (0.25%)	1 (4.76%)

**Table 3:** Molecular and clinicopathological description of germline mutations

*Gene*	Variant (HGVS nomenclature)	AF exomes (gnomAD v2.1.1)	AF latino (gnomAD v2.1.1)	AF population of study	ACMG/AMP classification	ENIGMA classification	SNP ID	Molecular subtype	Personal history of cancer	Family history of cancer	Number of patients
*BRCA1*	NM_007294.3:c.5123C > A (p.Ala1708Glu)	0.00001990	0.00005784	0.00125	NA	Pathogenic (BRCAexchange)	rs28897696	TNBC	No	No	1
*BRCA1*	NM_007294.3:c.5324 T > G (p.Met1775Arg)	0.00001193	0	0.00125	NA	Pathogenic (BRCAexchange)	rs41293463	ER-,PR + ,HER2 +	No	No	1
*BRCA1*	NM_007294.3:c.1674delA (p.Gly559Valfs*13)	NR	NR	0.00125	NA	Pathogenic (BRCAexchange)	rs80357600	TNBC	No	Yes	1
*BRCA2*	NM_000059.3:c.2380dupA (p.Met794Asnfs*8)	NR	NR	0.00125	NA	Pathogenic (BRCAexchange)	rs730881602	ER + ,PR-,HER2-	No	No	1
*BRCA2*	NM_000059.3:c.3860delA (p.Asn1287Ilefs*6)	0.0000153412	0	0.00125	NA	Pathogenic (BRCAexchange)	rs80359406	ER-,PR-,HER2 +	No	Yes	1
*BRCA2*	NM_000059.3:c.4889C > G (p.Ser1630Ter)	0.00000803232	0	0.00125	NA	Pathogenic (BRCAexchange)	rs80358711	ER-,PR + ,HER2-	No	Yes	1
*BRCA2*	NM_000059.3:c.5773C > T (p.Gln1925Ter)	0.00000398902	0	0.00125	NA	Pathogenic (BRCAexchange)	rs80358806	ER + ,PR + ,HER2-	No	Yes	1
*BRCA2*	NM_000059.3:c.5851_5854delAGTT (p.Ser1951Trpfs*11)	NR	NR	0.00125	NA	Pathogenic (BRCAexchange)	rs80359543	ER + ,PR + ,HER2 +	Yes	No	1
*BRCA2*	NM_000059.3:c.2808_2811delACAA (p.Ala938Profs*21)	0.00000797283	0	0.0025	NA	Pathogenic (BRCAexchange)	rs80359351	ER + ,PR + ,HER2-; ER + ,PR + ,HER2-	No; No	Yes; Yes	2
*BRCA2*	NM_000059.4:c.1763_1766delATAA (p.Asn588Serfs*25)	NR	NR	0.0025	NA	Pathogenic (BRCAexchange)	rs80359303	ER + ,PR + ,HER2-; TNBC	No; Yes	Yes; Yes	2
*BRCA2*	NM_000059.4:c.9097dupA (p.Thr3033Asnfs*11)	NR	0	0.00125	NA	Pathogenic (BRCAexchange)	rs397507419	ER + ,PR + ,HER2-	No	No	1
*ATM*	NM_000051.3:c.4507C > T (p.Gln1503Ter)	0.00000795817	0.00005783	0.00125	Pathogenic (PVS1 + PS4 moderate + PM2 supporting)	NA	rs2227945	ER + ,PR + ,HER2-	No	No	1
*ATM*	NM_000051.3:c.3510dupA (p.Gln1171Thrfs*8)	0.00000397874	0.00002891	0.00125	Pathogenic (PVS1 + PS4 supporting + PM2)	NA	rs876658899	ER + ,PR + ,HER2-	No	Yes	1
*ATM*	NM_000051.3:c.5496 + 2_5496 + 5delTAAG	NR	NR	0.00375	Likely pathogenic (PVS1 moderate + PS4 moderate + PM2)	NA	rs1565479572	ER + ,PR + ,HER2-; ER + ,PR + ,HER2-; ER + ,PR-,HER2-	No; Yes; No	No; Yes; Yes	3
*BARD1*	NM_000465.3:c.176_177delAG (p.Glu59Alafs*8)	NR	NR	0.00125	Pathogenic (PVS1 + PM1 + PM2)	NA	rs1057517589	TNBC	No	No	1
*CHEK2*	NM_007194.3:c.1100delC (p.Thr367Metfs*15)	0.00204432	0.00005649	0.00125	Pathogenic (PVS1 + PS3 + PS4 moderate + PM1)	NA	rs555607708	ER + ,PR + ,HER2 +	No	Yes	1
*CHEK2*	NM_007194.3:c.349A > G (p.Arg117Gly)	0.00011941	0.0001129	0.00125	Likely pathogenic (PS3 + PS4 moderate + PM1 + PP3)	NA	rs28909982	ER + ,PR + ,HER2-	No	Yes	1
*PALB2*	NM_024675.3:c.984delT (p.Leu329Terfs)	NR	NR	0.00125	Pathogenic (PVS1 + PM2)	NA	Novel	ER + ,PR + ,HER2 +	No	No	1*
*PALB2*	NM_024675.3:c.986delT (p.Leu329Glnfs*17)	NR	NR	0.00125	Pathogenic (PVS1 + PM2)	NA	Novel	ER + ,PR + ,HER2 +	No	No	1*
*PALB2*	NM_024675.3:c.3350 + 4A > G	0.000003977	0	0.00125	Likely pathogenic (PS3 very strong + PM2 + BP4)	NA	rs180177136	TNBC	No	Yes	1
*RAD51D*	NM_002878.3:c.556C > T (p.Arg186Ter)	0.00004011	0	0.00125	Pathogenic (PVS1 + PS4 supporting + PM1)	NA	rs387906843	ER + ,PR + HER2-	No	No	1

*NR* Not reported, *NA* Not applied, *variants identified in the same patient.

Estrogen and/or progesterone positive hormonal receptors (HR) were identified in 75% (18/24) of unselected women carrying P/LP variants. From them, germinal mutations distribution was: *BRCA2*, 44.4% (8/18); *ATM*, 27.8% (5/18); *CHEK2*, 11.1% (2/18); *BRCA1*, 5.5% (1/18); *PALB2* 5.5% (1/18) and *RAD51D*, 5.5% (1/18).

P/LP variants were found in 13/24 (54.2%) women without HER-2 amplification (independently of HR status), and their gene distribution was: *BRCA2*, 46.2% (6/13); *ATM*, 38.5% (5/13); *CHEK2*, 7.7% (1/13) and *RAD51D*, 7.7% (1/13). In contrast, oncoprotein HER-2 amplification (independently of HR status) was detected in 20,8% of the tumors. 40% (2/5) of the P/LP variants were identified in *BRCA2* gene and 60% (3/5) in *BRCA1*, *CHEK2* and *PALB2* genes.

TNBC tumors (according to the absence of HR expression and HER-2 amplification) were identified in 5/24 affected women (20.8%), from them, 40% (2/5) of the variants were detected in *BRCA1* gene, and 60% (3/5) in *BRCA2*, *BARD1,* and *PALB2* genes.

Ductal BC was present in 95.8% of the patients carrying a P/LP variant; metaplastic BC was observed in 1/24 (4.2%) women with a heterozygous *PALB2* gene mutation.

Interestingly, two patients (2/24) suffered more than one primary cancer. One woman was diagnosed with lymphoma with a prior ductal BC, and she had a heterozygous *BRCA2* c.1763_1766delATAA, p.Asn588Serfs*25; the other one had three cancers, thyroid, gastric, and ductal BC harboring an *ATM* c.5496 + 2_5496 + 5delTAAG, a likely pathogenic mutation.

Family history of cancer was documented in 14/24 women (58.3%), in their first, second, and third consanguinity-degree relatives. *BRCA2* accounts for 50% (7/14), with P/LP variants including a variety of tumors in relatives such as breast, thyroid, bone, gastric, prostate, leukemia, and esophagus, followed by *ATM*, in 3/14 (21.4%), which referred on thyroid, gastric, and brain cancers. Two women (14.3%) have P/LP variants in the *CHEK2* gene, and their relatives have breast and pancreas cancer. One patient (1/14) who had a pathogenic variant in the *BRCA1* gene, had several relatives with BC. Lastly, an affected woman with the *PALB2* gene intronic variant, described relatives with ovary, breast, and thyroid cancers.

Three recurrent germline mutations were detected: two in *BRCA2* (c.2808_2811delACAA, p.Ala938Profs*21 and c.1763_1766delATAA, p.Asn588Serfs*25); the other one in *ATM* (c.5496 + 2_5496 + 5delTAAG). From them, *BRCA2* c.1763_1766delATAA, p.Asn588Serfs*25, and *ATM* c.5496 + 2_5496 + 5delTAAG were not previously reported in the gnomAD v2.1.1 database (https://gnomad.broadinstitute.org/).

Regarding MLPA analysis for *BRCA1*/*2* genes, there were no large genomic rearrangements (LGRs) in the sample of 400 unselected women with BC.

### Correlation between mutation status and baseline characteristics of women with unselected BC

For women harboring germline mutations, statistical association tests were performed, comparing baseline characteristics of the population of study among 3 groups based on mutation status, such as absence of germline mutations (no mut), presence of germline mutations in *BRCA1/2* genes (BRCA), and germline mutations in no*BRCA* genes (*ATM, BARD1, CHEK2, PALB2,* and *RAD51D*) (Table 4). Women with germline mutations in *BRCA1/2* genes had an earlier age at diagnosis in comparison with no mut group (median age 36 *vs* 54, *p* = 0.0003), and 15.38% of women in the group BRCA had menopause in contrast with the no mut group were 57.68% of the patients had menopause (*p* = 0.009). The variable nodal stage (specifically nodal stage 2) had a higher frequency in the BRCA group (30.77% *vs* 9.26%, *p* = 0.0425) showing an association with the spread of cancer to a higher number of lymph nodes in this particular mutation status. No association with statistical significance was established for the no*BRCA* group.

**Table 4:** *BRCA* and no*BRCA* mutations statistical association test with clinical and pathological data

		*No mut*	*BRCA*	*noBRCA*	*BRCA vs No mut*	*noBRCA vs No mut*	*p-value*
*Age at diagnosis*		54 (43–65)	36 (30–44)	51 (45–56)	− 15.54 (− 20.92;− 10.16)	− 3.54 (− 9.1; 2.02)	**0.0003**
*Tumor size*		20 (12–30)	20 (15–25)	30 (16–39.5)	1.44 (− 14.61;17.49)	5.81 (− 7.41;19.02)	0.5662
*Positive nodes*		0 (0–1)	0 (0–1)	1 (0.25–4.25)	− 0.66 (− 1.07;-0.25)	2.71 (− 0.66;6.07)	0.0469
*Ki67*		25 (12–43.8)	35 (20–65)	25 (15–45)	8.59 (− 5.57;22.76)	2.77 (− 11.87;17.23)	0.3644
*Histologic diagnosis*	Ductal	319 (85, 29%)	13 (100%)	10 (90.91%)	–	–	0.5784
	Lobular	21 (5.61%)	0	0	0 (0.03;9.58)	0 (0.04;12.50)	
	Other	34 (9.09%)	0	1 (9.09%)	0 (0.02;5.90)	0.83 (0.23;7.59)	
*ER status*	Pos	291 (78.44%)	8 (61.54%)	9 (81.82%)	0.36 (0.14;1.28)	0.82 (0.25;4.32)	0.3353
*PR status*	Pos	261 (70.35%)	9 (69.23%)	8 (72.73%)	0.76 (0.28;2.80)	0.84 (0.29;3.63)	0.9814
*HER-2 status*	Pos	91 (24.66%)	2 (15.38%)	2 (18.18%)	0.50 (0.16;2.65)	0.60 (0.19;3.29)	0.8696
*TNBC status*	Yes	41 (11.05%)	3 (23.08%)	2 (18.18%)	2.14 (0.76;9.28)	1.57 (0.50;8.76)	0.1744
*Nodal stage*	0	195 (53.13%)	6 (46.15%)	3 (27.27%)	–	–	**0.0425**
	1	126 (34.33%)	3 (23.08%)	5 (45.45%)	0.66 (0.22;3.11)	1.92 (0.62;9.45)	
	2	34 (9.26%)	4 (30.77%)	1 (9.09%)	3.18 (1.12;13.77)	1.39 (0.35;17.01)	
	3	12 (3.27%)	0	2 (18.18%)	0 (0.06;22.59)	7.5 (2.00;62.44)	
*Tumoral stage*	I	88 (23.78%)	3 (23.08%)	0	-	-	0.3523
	II	171 (46.22%)	5 (38.46%)	7 (63.64%)	0.64 (0.21;3.17)	3.58 (0.44;137.09)	
	III	96 (25.95%)	5 (38.46%)	3 (27.27%)	1.13 (0.37;5.68)	2.72 (0.33;126.04)	
	IV	15 (4.05%)	0	1 (9.09%)	0 (0.04;16.58)	5.5 (0.67;439.91)	
*Age of menarche*		13 (12–14)	14 (13–15)	13 (11–13.5)	0.95 (− 0.50;2.40)	− 0.75 (− 2.28;0.80)	0.2672
*Parity*		2 (2–3)	2 (2–3)	2 (1–2.75)	− 0.18 (− 0.76;0.39)	− 0.64 (− 1.31;0.04)	0.4164
*Born children*		2 (2–3)	2 (1.5–2)	1.5 (1–2.75)	− 0.56 (− 1.01;-0.11)	− 0.57 (− 1.27;0.13)	0.2465
*Age first born child*		23 (19–28)	20 (18.5–23.5)	26 (21.5–33.5)	− 2.95 (− 5.63;-0.27)	2.59 (− 1.44;6.63)	0.1001
*Lactation*	Yes	301 (80.48%)	11 (84.62%)	10 (90.91%)	0.89 (0.30;4.51)	1.21 (0.30;9.62)	0.6466
*Menopause*	Yes	214 (57.68%)	2 (15.38%)	6 (54.55%)	0.12 (0.04;0.64)	0.73 (0.27;2.75)	**0.009**
*Age of menopause*		50 (46–52)	52.5 (51.75–53.25)	50.5 (46–54.25)	3.27 (0.26;6.28)	1.27 (− 3.21;5.75)	0.4365
*Weight*		65 (58–73)	68 (57–75)	73.3 (65.8–79.5)	2.95 (− 5;10.9)	6.07 (-0.05;12.2)	0.1377
*Height*		158 (155–163)	164 (157–165)	158 (151.5–160)	3.25 (− 0.35;6.84)	− 2.25 (− 5.76;1.26)	0.1143
*BMI*		25.84 (23.44–29.02)	25.33 (24.14–29)	29.4 (25.96–33.95)	− 0.07 (− 2.33;2.20)	3.14 (0.71;5.57)	0.0666
*Overweight*	Yes	226 (60.59%)	7 (53.85%)	8 (72.73%)	0.65 (0.26;2.19)	1.29 (0.45;5.60)	0.6297
*Hormonal contraception*	Yes	186 (49.87%)	5 (38.46%)	5 (45.45%)	0.64 (0.18;1.98)	0.84 (0.23;2.92)	0.8011
*HRT*	Yes	24 (6.45%)	0	0	0 (0.03;9.13)	0 (0.03;10.81)	1
*Smoking*	Yes	95 (25.40%)	3 (23.08%)	4 (36.36%)	0.79 (0.28;3.34)	1.45 (0.53;5.78)	0.6582
*Alcohol consumption*	Yes	47 (12.60%)	2 (15.38%)	1 (9.09%)	1.13 (0.37;6.07)	0.62 (0.17;5.58)	0.8771
*Radiation exposure*	Yes	23 (6.61%)	1 (8.33%)	1 (9,09%)	1.13 (0.31;10.42)	1.23 (0.34;11.52)	0.4583
*Personal history cancer*	Yes	19 (5.08%)	1 (7.69%)	1 (9.09%)	1.36 (0.38;12.64)	1.61 (0.44;15.30)	0.3868
*Family history cancer*	Yes	260 (70.08%)	9 (69.23%)	6 (54.55%)	0.77 (0.29;2.83)	0.42 (0.16;1.61)	0.5433
*NCCN criteria v1.2023*	Yes	224 (59.89%)	11 (84.62%)	8 (72.73%)	2.44 (0.77;12.30)	1.33 (0.46;5.75)	0.1439
*Inheritance*	Sporad	148 (39.89%)	5 (38.46%)	5 (45.45%)	-	-	0.8491
	Fam	175 (47.17%)	6 (46.15%)	6 (54.55%)	0.84 (0.32;3.18)	0.84 (0.32;3.18)	
	Her	48 (12.94%)	2 (15.38%)	0	1.01 (0.30;6.43)	0 (0.02;5.13)	

Qualitative variables are summarized as frequencies and percentages, and quantitative variables are reported as medians and interquartile ranges. Effect measure and IC95% were determined with means difference and OR (odds ratio) for quantitative and qualitative variables, respectively. *P*-values calculated using the Kruskal-Wallis test for quantitative variables and the Chi-square test for qualitative variables. Associations with statistical significance are shown in bold (*p*-value < 0.05). sporad: sporadic fam: familial her: hereditary

### Segregation analysis

Analysis was performed in six families which were ascertained by an index case: three families for three different pathogenic variants in the *BRCA2* gene, two with a likely pathogenic variant in the *ATM* gene, and one family with a likely pathogenic variant in the *PALB2* gene (Fig. 1). In total, 13 relatives tested positive for the mutations assessed (11 in *BRCA2* and two in *ATM*). Particularly, two *BRCA2* families with a pathogenic variant were tested, one of the relatives had been diagnosed with BC at 41 years (c.1763_1766delATAA, p.Asn588Serfs*25; age diagnosis index case: 35 years), and another relative was diagnosed with breast and thyroid cancer at 64 years (c.3860delA, p.Asn1287Ilefs*6; age diagnosis index case: 42 years). All of the relatives who tested positive received genetic counseling.

### Minigene assay

**Fig. 2:**
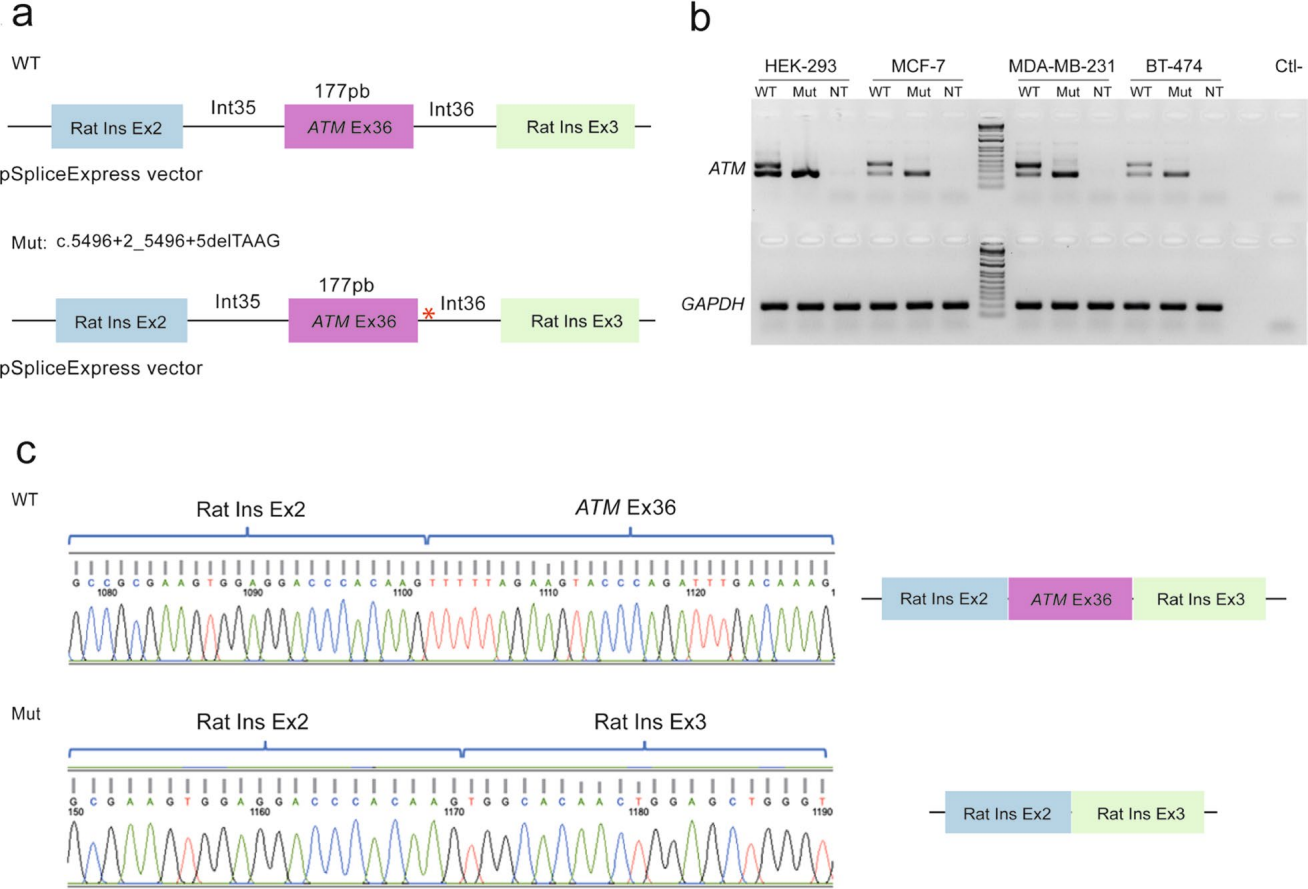
Exon skipping of exon 36 of the *ATM* gene due to germline mutation c.5496 + 2_5496 + 5delTAAG. **a** Diagram of the minigene pSpliceExpress vectors, WT which is constituted by exon 36 of *ATM* and exons 2 and 3 from Rat insulin (Rat Ins Ex2 and Rat Ins Ex3), and Mut which represents the presence of the germline mutation of interest. **b** RT-PCR, performed after transfection of the WT and Mut plasmids, in HEK-293, MCF-7, MDA-MB-231, and BT-474 cell lines, showed exon skipping in all cell lines, negative control was not transfected cells (NT). **c** Sanger sequencing was performed to confirm the effect in splicing observed in RT-PCR

Three affected and unrelated women showed heterozygous *ATM* c.5496 + 2_5496 + 5delTAAG variant. A minigene assay was performed to identify the alternative splicing effect in mRNA. This assay evidenced an exon 36 skipping which was confirmed by Sanger sequencing (Fig. 2).

## Discussion

Identifying germline mutations in high and moderate-risk BC genes is of paramount importance for establishing genetic screening programs that facilitate early diagnosis and development of national public health policies. Implementation of genomic analysis through NGS and incorporation of no*BRCA* genes has proven to be an adequate strategy to increase sensibility regarding recurrent mutation analysis restricted only to *BRCA1/2* genes [12, 13].

Globally, germline mutation cancer prevalence, can be estimated from hereditary, familial, or unselected BC cases. European, North American, and Asian populations have been the primary focus to obtain this data.

To our knowledge, this is the first report on the prevalence of mutations in the top 10 clinically impactful genes, identified by WES in 400 women with unselected BC from various regions of Colombia.

We evaluated NCCN criteria [10] in the women studied. Significantly, 20.8% of them with a P/LP variant did not fulfill those criteria. This finding demonstrates that molecular testing should be considered in all women with BC regardless of the age of diagnosis, molecular subtype, and personal or family history of cancer.

Our findings determined that 6% of the Colombian women with unselected BC had germline mutations in seven of the genes studied, being *BRCA2* the gene with the highest frequency of variants and women affected, followed by *ATM, BRCA1, PALB2, CHEK2, BARD1* and *RAD51D* genes. No P/LP variants were detected in *CDH1, RAD51C,* and *TP53* genes. BC prevalence of germline mutations and their frequency in cancer risk genes, varies thoroughly depending on the selection criteria of the population studied.

Interestingly, to date at least, 41 articles have been described that analyze genes related to BC in the Latin American population. This includes a diversity of patients from Argentina, Brazil, Chile, Guatemala, Colombia, Peru, Puerto Rico, and Mexico, covering 40% of the countries considered in the region through genetic analysis (Supplementary Table 1, and the references therein). These studies have examined approximately 51,000 Latin American patients, which have provided insights into the frequencies of molecular variants of interest in the analyzed genes (*BRCA1/2* and no*BRCA*) (Supplementary Table 1 and the references therein). Concerning the mutational spectrum exhibited by the *BRCA1/2* genes, a range from 10.1% to 37.2% has been noted across the populations. This variation is estimated to be strongly linked to the migration history of the Latin American populations, including the overlap of some mutations determined by shared events and exchanges that characterize the migration history of each geographical region [14]. Additionally, within the same population, such as Brazil, there is high variability in the mutation frequencies of the *BRCA* genes (10.1% vs 22.4%), supporting the observation that the genetic background of Latin American populations results from events leading to unique population structures within and between countries [14–16]. Specifically, the highest frequencies for the *BRCA* genes reported in the Latin American population are described in patients with breast and ovarian cancer from Afro-Colombian families, in whom 33.3% of pathogenic variants were identified [17], demonstrating the impact of patient selection criteria on the variability of reported data.

Unlike our study, most studies reported in Latin America have involved patients with hereditary BC, in whom the representation of pathogenic variants in the *BRCA* genes is substantially higher than in cases of unselected BC. For this latter group, frequencies between 1.2 and 14.5% have been reported (with eight studies in Latin America) [6, 18–24], which is consistent with the findings identified in the present study (Table 2 and supplementary Table 1). The analysis of unselected populations has been recommended to avoid the overestimation of the true prevalence of germline cancer-related P/LP variants in the general population [25].

In Colombia, previous reports described mutations in *BRCA1/2* genes focused on hereditary/familial cases [17, 21, 26, 27]. Even though, few studies analyzed mutation prevalence in *BRCA1/*2 genes from unselected BC patients, finding that their frequency ranges from 0.4 to 3.3% [20, 21], which is concordant with our results, since the frequency of women with mutations in *BRCA1/2* is 3.25%.

Beyond *BRCA* genes in Latin America NGS multigene analysis has been conducted in 78% of studies, including the current study, which has enabled the identification of P/LP variants in moderate and low cancer-risk genes, potentially actionable [28]. Our study demonstrated that while 52.3% of the P/LP variants were associated with *BRCA1/2* genes, nearly 50% of the women had mutations in no*BRCA* genes. These findings are similar to those reported in unselected BC populations from countries such as Argentina and Guatemala, where the contribution of no*BRCA* genes was described as 4.7% and 3.2%, respectively [6, 19]. Similar to studies concerning hereditary BC cases, the frequency of P/LP mutations in no*BRCA* genes constitutes a significant proportion (Supplementary Table 1). Paixão et al. (2022) found P/LP variants from 9.6% *(BRCA1/2*) to 25.2% (no*BRCA*) analyzing 321 Brazilian patients with a panel of 94 genes [7]. Additionally, Cock-Rada and colleagues assessed 25 cancer susceptibility genes in 85 women from Medellin, who met the criteria for HBOC molecular testing; this study identified mutations in six genes: *BRCA2, BRCA1, PALB2, ATM, MSH2*, and *PMS2* [29]. All these findings describe germline mutation profiles which, like our results, demonstrate the contribution to the genetic variability in BC of genes such as *ATM, PALB2*, and *CHEK2*, and should be taken into consideration. This finding is consistent with reports from other Latin American populations, where mutations in *PALB2* or *RAD51C* explain a significant proportion of cases. The present results, along with others previously published, demonstrate that the analysis of genes other than *BRCA1/2* increases the detection rate of P/LP variants, which maximizes the identification of germline variants in patients with hereditary and unselected BC.

In our study, we identified recurrent mutations in 1.75% of the population analyzed, indicating that most P/LP variants are private. This finding is consistent with previous reports in other LATAM populations, where the recurrence of mutations is low [13]. Three recurrent variants were identified: two in the *BRCA2* gene (c.2808_2811delACAA, p.Ala938Profs*21 and c.1763_1766delATAA, p.Asn588Serfs*25), and one in the *ATM* gene (c.5496 + 2_5496 + 5delTAAG). Two women carrying recurrent mutations in *BRCA2* (c.1763_1766delATAA, p.Asn588Serfs*25) and *ATM* (c.5496 + 2_5496 + 5delTAAG) genes had the diagnosis of other types of cancer that is, lymphoma and, thyroid and gastric, respectively. Co-occurrence between BC and other types of cancer has been pinpointed in the literature [30, 31]. Specifically, P/LP variants in the *ATM* gene are associated with gastric and thyroid cancers, and risk estimates have also been described; for gastric cancer, several studies associated *ATM* mutations with OR (odds ratio) ranging from 2.97 to 4.74 [32–34]. Recently, the association between *H. pylori* infection and germline P variants in genes as *BRCA1, BRCA2, ATM,* and *PALB2*, has been described; people with *H. pylori* infection and germline mutations in those genes have a higher gastric cancer cumulative risk at 85 years of 45.5% (95% CI, 20.7 to 62.6); in contrast, the risk in people with *H. pylori* infection alone is 14.4% (95% CI, 12.2 to 16.6) [35]. Thyroid cancer (TC) has also been associated with the presence of germline mutations in *BRCA2* and *ATM* genes [36, 37]. Interestingly, a published study showed an increased oncogenic SNPs burden in cases with co-occurrence of BC and TC. In patients with double cancers, germline variants were found in *PALB2, BRCA1, BRCA2, ATM,* and *CHEK2* genes, which are known risk genes associated with BC [38].

Recurrent variants could also be considered founder mutations. The prevalence of founder mutations has been extensively documented for the *BRCA1* and *BRCA2* genes. These pathogenic variants represent the majority of observed mutations in specific populations and have been confirmed as true founders through analysis of common ancestral haplotypes [39]. In our population of study three Colombian founder mutations, previously described [21], were identified, one in *BRCA1* c.5123C > A (A1708E), and two in *BRCA2* c.1763_1766delATAA (1991del4) and c.2808_2811delACAA (3034del4).

Identification of recurrent pathogenic variants in the *ATM* gene is of importance, as previous studies have demonstrated that women carrying mutations in this gene have a significantly increased risk of developing BC with a risk similar to that conferred by germline mutations in the *BRCA2* gene [40]. Interestingly, the allelic frequency of the *ATM* variant c.5496 + 2_5496 + 5delTAAG was 0.375%, although it has not been previously reported in the population database gnomAD, the variant has been identified in cases related to ataxia-telangiectasia syndrome, familial breast cancer, and hereditary cancer predisposition syndrome. These findings are not supported by population-based studies but have been submitted by molecular diagnostic centers such as Color Diagnostics (2019), Fulgent Genetics (2021), Baylor Genetics (2022), Invitae (2022), Ambry Genetics (2023), and Myriad Genetics (2024) (https://www.ncbi.nlm.nih.gov/clinvar/variation/VCV000565770.15 (accessed May 7, 2024)). In all instances, the variant has been determined to be germline. However, due to the unknown origin, the number of affected individuals, or the lack of familial segregation analysis, we cannot make comparisons with the data from the current study. It is noteworthy to date, this variant has been attributed  to a significant impact on RNA splicing, although this has not been experimentally proven, hence its classification according to ACMG criteria is likely pathogenic. Functional validation of this recurrent variant demonstrated an exon skipping, leading to a predicted deletion of 59 amino acids located in the Pincer domain of the ATM protein [41]. The splicing process is an event that most eukaryotes genes go through and is regulated by RNA-Binding Proteins (RBPs), *cis*-regulatory elements, and *trans*-acting factors [42]. Alternative splicing is dysregulated in cancerous cells in comparison with healthy cells, and carcinogenesis has been associated with alterations in direct and indirect regulators, leading to altered splicing profiles [43]. In the present study, the minigene assay resulted in an exon skipping, caused by a *cis*-regulatory element (c.5496 + 2_5496 + 5delTAAG) on the *ATM* gene. This molecular finding added to the absence of this mutation in the gnomAD database, supports the pathogenic effect of the mutation in the function of the ATM protein and the possible role in BC development. Dysregulation of alternative splicing in cancer has made it a therapeutic target and several therapeutic strategies are currently under study; that is, targeting RNA splicing factors, splicing factors regulated by blocking kinases, and antiRNA molecules [44].

Although 58.3% of women with a mutation had several relatives with various types of cancer, segregation analysis was performed in some families with index cases having P/LP variants in *BRCA2, ATM,* and *PALB2* genes. Interestingly, segregation of P/LP variant and phenotype was observed in two families tested for *BRCA2* mutations (Fig. 1). Index cases of these families had an earlier age of onset compared with their relatives who suffered BC as well, suggesting anticipation phenomena. This finding may be associated with the greater penetrance of the *BRCA1/2* genes, compared to other genes with moderate penetrance such as *ATM*, but some authors have proposed the interference of non-genetic factors as an explanation for this anticipation [45, 46].

Germline pathogenic small indels and LGRs contribute to the development of breast and ovarian cancers [47]. Ratios of *BRCA1/2* LGRs germline mutations are population dependent [22, 48–56]. To our knowledge, in Colombia, *BRCA1/2* LGRs have been tested in two studies. Vargas and colleagues tested 60 Afro-Colombian families with HBOC, they did not find LGRs in that population [17]. Torres and colleagues tested 221 breast/ovarian cancer families, finding a LGR in the *BRCA2* (ex1-14del) gene in two unrelated patients (0,9%) [21]. Considering the three Colombian cohorts of patients assessed for *BRCA1/2* LGRs (Vargas et al., Torres et al., and ours), the prevalence of this type of rearrangement in *BRCA1/*2 genes would be 0,3% (2/681). Pondering the frequencies described previously, LGRs prevalence in *BRCA1/2* genes is low in Colombian BC patients, regardless of hereditary or family history.

This study has some limitations. The germline variants analyzed are rare and although they are located in high and moderate-risks genes, common SNPs also contribute to the development of BC. LGRs were only studied in *BRCA1/2*, although this type of rearrangement has been found in genes including *CHEK2* and *ATM*, in BC patients [57].

In conclusion, molecular analysis via WES enabled the establishment of the genomic profile of P/LP variants in ten clinically significant genes related to BC risk in the analyzed population. Additionally, this investigation was conducted in a population of women with unselected BC, which has been less addressed in the global literature compared to the vast amount of research conducted on individuals with hereditary cancer. Based on the information described and our study results, the germline mutation profile exhibits variation in genes and frequencies, contingent upon the region and characteristics of the population assessed. This underscores the importance of conducting population-based studies and determining the prevalence of clinically impactful genes. Such efforts can aid in the identification of mutations and facilitate the implementation of national genetic analysis policies, genetic counseling, and early detection strategies. Our study also highlights the utility of WES as an appropriate method for identifying germline variants located in coding and exon–intron boundary regions of genes that are clinically relevant in BC. WES analysis has the potential to detect rare, novel, and infrequently studied P/LP variants, including intronic mutations.

### Supplementary Information

Supplementary material 1.

## Data Availability

Further data and the datasets supporting this study are available from the corresponding author upon justified demand.

## Abbreviations

ACMG/AMP: American College of Medical Genetics and Genomics/American Molecular Pathology
BC: Breast cancer
BMI: Body mass index (kg/m^2^)
ER: Estrogen receptor
HBOC: Hereditary breast and ovary cancer
HER-2: Human epidermal growth factor receptor 2
HR: Hormonal receptors
HRT: Hormonal replacement therapy
LATAM: Latin-American
LGRs: Large genomic rearrangements
MLPA: Multiplex ligation dependant probe amplification
NCCN: National cancer comprehensive network
NGS: Next generation sequencing
P/LP: Pathogenic/likely pathogenic
PR: Progesterone receptor
SNP: Single nucleotide polymorphism
TNBC: Triple negative breast cancer
WES: Whole exome sequencing
