# Supplementary material for: Germline mutations of breast cancer susceptibility genes through expanded genetic analysis in unselected Colombian patients
Source: Hum Genomics. 2024 Jun 18;18:68. doi: 10.1186/s40246-024-00623-7 (PMC11184794; doi:10.1186/s40246-024-00623-7)
Supplement: Supplementary file 1 — Supplementary material 1. [file 40246_2024_623_MOESM1_ESM.docx]

**Germline mutations of breast cancer susceptibility genes through expanded genetic analysis in unselected Colombian patients**

**Supplementary methods**

**Clinical Data Collection.**

The clinical and sociodemographic variables of the patients were collected using a standardized format of medical history and structured interviews conducted by specialized physicians, this data included past medical history, family history, and data about tumor phenotype that was extracted from pathology reports. A 3-generation structured pedigree was constructed by a genetic counselor.

**MLPA (*Multiplex Ligation-dependent Probe Amplification* – MLPA).**

Probemix P002-D1-BRCA1 and P090-C1-BRCA2 contain 48 and 50 MLPA probes respectively were used according to the manufacturer´s instructions (<https://www.mrcholland.com>). The 3500 Genetic Analyzer was used to separate the amplified products by capillary electrophoresis using GeneScan350 ROX as a standard internal lane size. Data analysis was performed with Coffalyser.Net software (<https://www.mrcholland.com/technology/software/coffalyser-net>). Dosage Quotient Distribution (DQs) (based on a large number of samples at MRC-Holland) was used to determine the patient genotype. For homozygous deletion, the DQ value was 0; for heterozygous deletion 0.40<DQ<0.65; Normal 0.80<DQ<1.20; heterozygous duplication 1.30<DQ<1.65; homozygous duplication 1.75<DQ<2.15; all other values were considered ambiguous results (<https://www.mrcholland.com>). Copy number variations detected with the P002-BRAC1 and P090-BRCA2 probemix were verified using the SALSA MLPA probemix P087 and P077 respectively. The ligation sites of confirmatory probes have a distance of at least 20nt from probe ligation sites of the diagnosis probemix.

**Next Generation Sequencing (NGS – whole exome sequencing WES).**

For 350 samples three micrograms aliquot of genomic DNA were sent to the Novogene Inc. platform (Beijing, China) for Whole Exome Sequencing (6Gb). Briefly, the DNA quality was assessed on 1% gel agarose, and concentration was measured using Qubit® DNA Assay Kit in Qubit® 2.0 Fluorometer (Life Technologies, CA, USA). One microgram was used to prepare the library using the Agilent SureSelect Human All Exon v6 kit (Agilent) following the manufacturer’s recommendations.

DNA was sonicated (Covaris, MA, USA) in fragments with size of 180-280bp and the remaining overhangs were converted into blunt ends by enzyme activity which are eliminated after the enzyme reaction. Adapter oligonucleotides were ligated after adenylating DNA fragment 3ʹ ends. After PCR reaction, the libraries were hybridized with biotin-labeled probes and captured by magnetic beads with streptavidin. After washing, the libraries were enriched in a PCR reaction to add index tags. Then the products were purified with AMPure XP system (Beckman Coulter, Beverly, USA) and Agilent high sensitivity DNA assay on the Agilent Bioanalyzer 2100 system was used to quantify them.

If the library is appointed as “PASS”, it will be sequenced on Illumina NovaSeq 6000 platform. The short reads were recorded in FASTQ format. Novogene performed a quality control and required a value of Q30 for above 80% of paired-end sequencing data. Burrows-Wheeler Aligner (BWA) was used to map the paired-end clean reads to the human reference genome (hg19) and GATK v3.8 was used to call the variants. The variant annotation was performed using ANNOVAR for location and predicted function. The previous methodology was taken from Novogene´s technical report.

The library of 50 additional samples was constructed from 250ng of DNA with the MGIEasy FS DNA Library Prep kit and DNA was fragmented by enzymes approximately 200-400bp. The fragmented DNA was repaired, amplified by PCR and captured by Exome Capture V5 Probe with streptavidin beads and specific primers were used to amplify and enrich the regions of interest. A circularization of the DNA was realized and then a denaturation of the library after ligation of Split oligo and digestion and purification by specific beads. The circulated DNA was used to generate nanoballs (DNB), which consist in a cyclic amplification through Rolling Circle Amplification process. DNB were quantified and charged into a flowcell (FCL_PE100) to be sequenced by DNBseqG400. After sequencing, the quality of data was 90% with Q30.

The reads were mapped to the reference genome hg19 using BWA (Burrows-Wheeler Aligner) and organized by SAMtools. Duplicated reads were eliminated by Picard software. Variants were identified with Haplotyper (Sentieron) software v4.0.5.1. Depth and breadth of coverage were analyzed by BAMBA software to achieve a depth of 50x. The previous methodology was taken from Gencell´s report.

**Bioinformatic analysis and germline variant classification.**

For 400 WES, molecular variants identified in the genes *BRCA1*, *BRCA2*, *ATM*, *PALB2*, *CHEK2, BARD1, RAD51C, RAD51D, CDH1* and *TP53,* which were filtered using the Golden Helix software (VarSeq v2.3.0), using VCF format, considering the following filtering criteria: missense, nonsense, frameshift, stop codon gain and intronic (+/-15pb flanking the exons) variants, with additional MAF ≤0.01 for exomes, genomes and Latino population frequencies data (<https://gnomad.broadinstitute.org>).

Variant pathogenicity classification was annotated according to the ACMG/AMP (American College of Medical Genetics and Genomics and the Association for Molecular Pathology – ACMG/AMP)(1), ClinGen guidelines (<https://clinicalgenome.org/working-groups/sequence-variant-interpretation/>)(1–8), and ENIGMA for *BRCA1* and *BRCA2* genes (Evidence-based Network for Interpretation of Germline Mutant Alleles – ENIGMA)(9) guidelines.

Sanger sequencing confirmation was performed to the variants classified as pathogenic or likely pathogenic by either of the guidelines.

**Functional validation of the recurrent intronic variant in *ATM* gene (minigene assay).**

Plasmid construction.

Using the genomic DNA obtained from a heterozygous patient carrying the intronic variant c.5496+2_5496+5delTAAG in the *ATM* gene, we amplified by standard PCR a genomic region that encompassed the exon 36 and ±300 bp upstream and downstream intronic sequences. The primers used contained the attB1 adapter F and attB2 adapter R recombination sites (H_ATM_Int35_pSpliceExpress_F: GGGGACAAGTTTGTACAAAAAAGCAGGCTcattttgaaatcaagtctctgtgg, H_ATM_Int36_pSpliceExpress_R: GGGGACCACTTTGTACAAGAAAGCTGGGTctcctttttcttattcaagaaagc). The fragments were amplified using Q5® High-Fidelity 2X Master Mix (New England Biolabs, Ipswich, MA, USA). PCR products were purified using 30% PEG 8000/30 mM MgCl2 Solution and TE buffer pH 8, following the kit protocol (Gateway® BP Clonase™ II Enzyme Mix, Invitrogen, Carlsbad, CA, USA). The quantity and quality of the purified PCR products were assessed by electrophoresis and through measurement of absorbance with a Nanodrop (OD260/280 and OD260/230).

Gateway BP clonase reaction was performed following the manufacturer’s recommendations of Gateway® BP Clonase™ II Enzyme Mix (Invitrogen, Carlsbad, CA, USA) we mixed 10–150 ng of the attB-PCR product to 150 ng of pSpliceExpress vector (Addgene, catalog #32485) in 10 μl of recombination reaction, to which was added 2 μl of the BP Clonase II enzyme mix and sufficient volume of TE buffer, pH 8.0, to complete the total reaction volume. The reaction was incubated at 25°C for 1 hour. Then 1 μl of Proteinase K Solution (2 µg/µl) was added to inactivate the enzymes. The samples were subsequently incubated at 37°C for 10 minutes to terminate the reaction and used to transform competent E. coli.

Transformation.

1 μl of Gateway BP clonase reaction was transformed into 50 μl of One Shot OmniMAX 2 T1 Phage-Resistant (T1R) Chemically Competent E. coli (Invitrogen, Carlsbad, CA, USA) according to manufacturer´s instructions. Transformed bacteria were plated on ampicillin-supplemented LB plates and incubated at 37°C overnight. Single colonies were inoculated in 5 ml of ampicillin-supplemented LB medium overnight, and their plasmid DNA was extracted by QIAprep Spin Miniprep Kit (QIAGEN, Germantown, MD, USA). Minigenes carrying WT or variant alleles were verified by Sanger sequencing (Macrogen, Seoul, South Korea).

Transfection and RT-PCR.

Positive clones carrying the DNA inserts (WT and mutant) were cultured in 100 ml of ampicillin-supplemented LB medium overnight and plasmidic DNA was extracted by QIAGEN Plasmid Midi Kit (QIAGEN, Germantown, MD, USA). The minigene constructs were transfected in four cell lines: HEK 293, MDA-MB-231, MCF-7, and BT-474 for 48 hours using Fugene 6 (Promega, Madison, WI, USA). Total RNA was extracted from cells using a TRIzol (Invitrogen) according to manufacturer´s instructions. RNA samples were treated with DNase I Amplification grade (Sigma-Aldrich, San Luis, MO, USA). cDNA was synthesized using the SuperScript III First–Strand Synthesis System with oligo [dT] primers, following the manufacturer’s instructions (Invitrogen). PCR was performed using GoTaq® Green Master Mix (Promega) according to the supplier’s protocol and the PCR products were analyzed on agarose gel (1.5%). Primers used for the PCR are located in the backbone of the pSpliceExpress vector (RatIns_Ex2_pSpliceExpress_For: GATCGATCCGCTTCCTGCCCC, RatIns_Ex3_pSpliceExpress_Rev: CTGCCGGGCCACCTCCAGTGCC). Each experiment was performed in triplicate for each cell line. RT-PCR products were extracted from the agarose gel using QIAquick Gel Extraction Kit (Qiagen). The amplicons were introduced into the PCR4-topoTA cloning vector (Invitrogen, Carlsbad, CA, USA), to enable confirmation of sequences through Sanger sequencing.

**Supplementary Figure 1:** Distribution of P/LP variants and type of variant for each gene in the sample of unselected women with breast cancer.


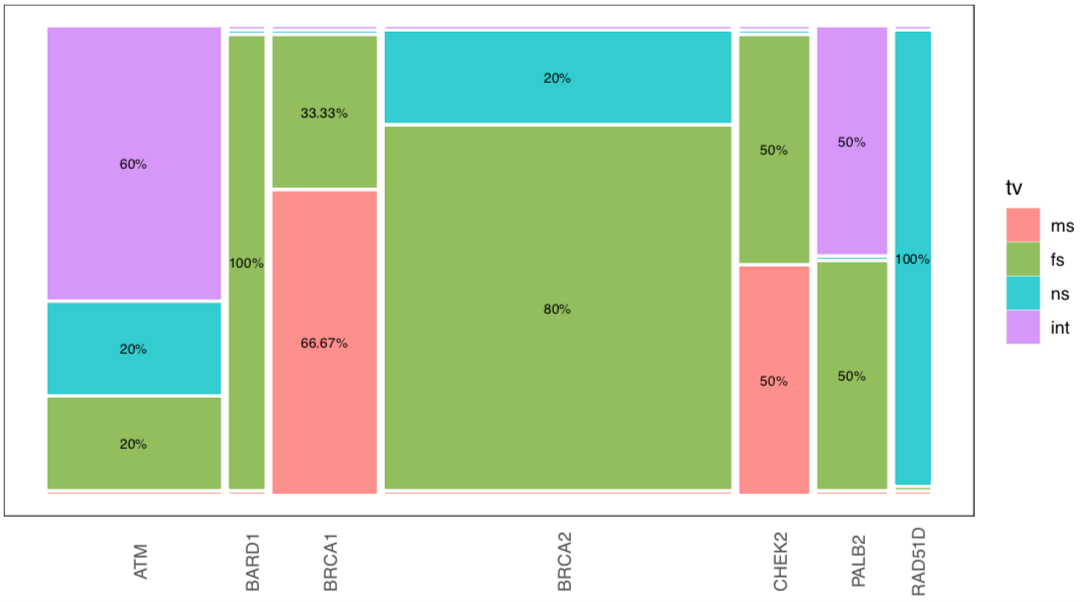


Mosaic plot. tv: type of variant, ms: missense, fs: frameshift, ns: nonsense, int: intronic

**Supplementary table 1**: Prevalence of *BRCA* and no*BRCA* P/LP germline variants in Latin-American population

| Country | Year | Population studied | Number of individuals analyzed | Genes analyzed | Overall prevalence of P/LP variants | Prevalence of BRCA P/LP variants | Prevalence of non-BRCA P/LP Variants | Reference |
| --- | --- | --- | --- | --- | --- | --- | --- | --- |
| Colombia | 2024 | Unselected women with BC | 400 | BRCA1, BRCA2, ATM, PALB2, CHEK2, BARD1, RAD51C, RAD51D, CDH1, and TP53 | 6% (24/400) | BRCA2: 2.5% (10/400); BRCA1: 0.75% (3/400) | 2.75% (11/400), genes: ATM, PALB2, CHEK2, BARD1, and RAD51D | Sierra-Díaz et al. |
| Argentina | 2018 | high risk individuals Ashkenazi Jewish | 279 | BRCA1 and BRCA2: Sanger sequencing for the recurrent variants (BRCA1: c.68_69del and c.5266dup; BRCA2: c.5496del). NGS and MLPA | Recurrent variants: 19.7% (55/279); non recurrent variants: 2.2% (6/279) | NA | NA | (10) |
|  | 2019 | unselected Argentinian BC patients | 112 | BRCA1, BRCA2, PALB2, and RAD51C | 10.7% (12/112) | 6.2% (7/112); BRCA2: 4.5% (5/112); BRCA1: 1.8% (2/112) | 4.7% (5/112); genes:PALB2 and RAD51C | (11) |
|  | 2021 | Patients with breast/ovary cancer (BOC), including TNBC | 2155 | BRCA1/2: 1900 patients. Multigene panel: ATM, BRCA1, BRCA2, BRIP1, CDH1, CHEK2, CDKN2A, EPCAM, MUTYH, NF1, NBN, PALB2, PMS2, PTEN, RAD51C, RAD51D, STK11, and TP53: 255 patients. | 17.2% (372/2155) | BRCA1: 9.6% (207/2155); BRCA2: 7.0% (152/2155) | 0.6% (13/2155); genes: CHEK2, MUTYH, CDH1, CDKN2A, NBN, RAD51D, and TP53: 0.05% (1/2155) | (12) |
|  | 2022 | Patients wtih clinical criteria for testing HBOC | 1905 | PALB2 | 2.78% (53/1905) | NA | 2.78% (53/1905) | (13) |
| Brazil | 2014 | Brazilian women who fulfilled criteria for HBOC | 120 | Comprehensive secreening of: BRCA1, BRCA2 (capillary sequencing and MLPA), TP53 R337H, CHEK2 c.1100delC (Capillary sequencing). Analysis of CNVs of the genes (array-CGH): PTEN, ATM, NBN. RAD50, RAD51, BRIP1, PALB2, MLH1, MSH2, MSH6, TP53, CDKN2A, CDH1, and CTNNB1 | 26% (31/120) | BRCA1: 16.6% (20/120); BRCA2: 5.8% (7/120) | 4.2% (5/120), genes: TP53, CHEK2 and ATM (heterozygous deletion of exon 4) | (14) |
|  | 2016 | HBOC; LFL; HBCC | 19; 40; 7 | BRCA1, BRCA2, TP53, and CHEK2 | NA | HBOC: BRCA2: 5.5% (1/18). | HBCC: 14.3% (1/7), gene: CHEK2 c.1100delC | (15) |
|  | 2018 | 132 patients with BC and 25 individuals unaffected with family history positive | 157 | Multi-gene panel | 14.6% (23/157) | BRCA1: 7% (11/157); BRCA2: 3.2% (5/157) | 4.4% (7/157), genes: ATM, ATR, CDH1, and MLH1 | (16) |
|  | 2020 | Individuals with clinical suspicion of HBOC syndrome in Southeeast Brazil | 94 | ABRAXAS1, ATM, ATR, BARD1, BRCA1, BRCA2, BRIP1, CDH1, CHEK2, MLH1, MRE11, MSH2, MSH6, NBN, PALB2, PMS2, PTEN, RAD50, RAD51, TP53, and UIMC1 | 23.4% (22/94) | BRCA1: 13.8% (13/94); BRCA2: 4.2% (4/94) | 5.3% (5/94), gene: TP53 | (17) |
|  | 2021 | Unrelated patients from Brasilia, with BC diagnosis | 224 | Multi-gene panel | 20.5% (46/224) | BRCA1: 5.8% (13/224); BRCA2: 2.7% (6/224) | 12.5% (28/224), genes: TP53, CHEK2, MUTYH, BARD1, RAD51C, MSH6, PALB2, ATM, RAD51D, RECQL4, and CTC1 | (18) |
|  | 2021 | Patients who fulfilled NCCN criteria and tested negative for BRCA1/2 | 126 | ATM, CDH1, CHEK2, PALB2, RAD51D, and TP53 | 3.97% (5/126) | NA | 3.97% (5/126), genes: CHEK2, ATM, PALB2, and TP53 | (19) |
|  | 2022 | Brazilian women referred to a single institution cancer risk assessment program. 173 women had invasive BC and 119 were cancer free women. | 292 | Breast cancer susceptibility genes: BROCA panel | 20.8% (36/173) | BRCA1: 6.9% (12/173), BRCA2: 5.8% (10/173) | 8.1% (14/173), genes: PALB2, TP53, ATM, BARD1, BRIP1, FAM175A, FANCM, NBN, and SLX4 | (20) |
|  | 2022 | Unrelated BC patients who fulfilled NCCN criteria for BRCA1/2 testing | 321 | 94-genes panel (TruSight Cancer Sequencing Panel) | 25.2% (81/321) | BRCA1: 5.3% (17/321); BRCA2: 4.4% (14/321) | 16.2% (52/321), genes: TP53, PALB2, ATM, CHEK2, RAD51C, MUTYH, SBDS, FANCI, HNF1A, PFR1, RECQL4, BLM, BRIP1, FANCA, FANCD2, FANCE, FANCL, FANCM, FH, PHOX2B, PMS2, and SLX4. | (21) |
|  | 2022 | Brazilian BC patients | 1663 | 20-38 cancer susceptibility genes | 20.1% (335/1663) | BRCA1: 5.8% (97/1663), BRCA2: 4.3% (72/1663). | 10% (166/1663), genes: TP53, MUTYH, ATM, CHEK2, PALB2, RAD51C, BRIP1, APC, BLM, FANCC, PMS2, RECQL, CDH1, NF1, PTEN, BARD1, RAD51D, MEN1, MSH2, and MLH1. | (22) |
| Chile | 2008 | 196 Patients with BC belonging to high risk Chilean families, 184/196 were BRCA1/2 negative. Control group: 500 healthy Chilean women with no personal or familiaL history of cancer. 624 healthy Chilean females but with at least two relatives in first or second degree with BC. | 1320 | CHEK2 c.1100delC. RFLP | None of the 1320 samples analyzed carried the variant. | NA | None of the 1320 samples analyzed carried the variant. | (23). |
|  | 2011 | Index cases belonging to high risk unrelated families, (mutation testing); high risk subjects, for hereditary BC and/or OC, (LGRs testing) | 326; 56 | BRCA1 and BRCA2 | 7.1% (23/326) | BRCA1: 3.4% (11/326); BRCA2: 3.7% (12/326). LGR analysis was negative. | NA | (24) |
|  | 2012 | Chilean BC cases negative for BRCA1/2 | 322 | BARD1 p.Cys557Ser | 2.5% (8/322) | NA | 2.5% (8/322) | (25) |
|  | 2015 | Chilean familial BC cases BRCA1/2 negative | 100 | PALB2 (conformational sensitive gel electrophoresis and direct sequencing) | NA | NA | No pathogenic variants were detected | (26) |
|  | 2019 | Individuals with personal or family history of breast and/or ovarian cancer. 105/315 were tested with genetic panels | 315 | BRCA1 and BRCA2. Genetic panels | 20.3% (64/315) | BRCA1: 8.2% (26/315); BRCA2: 8.9% (28/315) | 10.5% (11/105), genes: PALB2, CHEK2, CDH1, RAD51D, ATM, and RAD51C | (27) |
|  | 2024 | Individuals who underwent genetic counseling, 91.1% were women, and 75.2% had cancer. Of the patients with cancer, 96% were female, with breast and/or ovarian cancer (92.2%) | 722 | BRIP1, ABRAXAS1, AKT1, APC, ATM, AXIN2, BAP1, BARD1, BLM, BMPR1A, BRCA1, BRCA2, BRIP1, BUB1B, CDC73, CDH1, CDKN2A, CEP57, CHEK2, DICER1, DIL3L2, EPCAM, FANCC, FANCM, HOXB13, KIT, MEN1, MLH1, MRE11, MSH2, MSH6, MUTYH, NBN, NF1, NTHL1, PALB2, PDGFRA, PHOX2B, PMS2, POLD1, POLE, PTCH1, PTEN, RAD50, RAD51C, RECQL, RECQL4, RINT1, RNF43, RPS20, RUNX1, SDHB, SDHC, SMAD4, SMARCA4, STK11, TERT, TP53, TSC1, TSC2, VHL, WRN, and XRCC2 | 23.5% (170/722) | BRCA1: 7.3% (53/722), CNVs: 1% (7/722); BRCA2: 6.9% (50/722) | 9.3% (67/722), genes: APC, ATM, ATR, AXIN2, CDKN2A, CHEK2, FANCM, MSH2, MSH6, NBN, NF1, PALB2, PTEN, RAD50, RAD51C, RAD51D, RET, and TP53 | (28) |
| Clinical Cancer Genomics Community Research Network (CCGCRN) cohort | 2019 | Any individual receiving genetic cancer risk assessment at cancer centers and community-based clinics from 40 sites in the US and 5 sites in Latin America. 10000 women with history of BC | 10000: younger group (less than 65 years): 9412; Older group (65 years or more): 588 | All patients: BRCA1 and BRCA2. 318 individuals of older and 3493 of younger groups had (multigene panel test): CDH1, PALB2, PTEN, TP53, STK11, CHEK2, ATM, NF1, and NBN. 270 of older and 5919 of younger were only tested for BRCA1 and BRCA2. | Older group: 5.6% (33/588); younger group: 14.2% (1340/9412) | Older group: BRCA2: 2.4% (14/588); BRCA1: 1.7% (10/588). Younger group: BRCA1: 7.1% (666/9412); BRCA2: 5% (469/9412) | Older group: 3.1% (10/318), genes: CHEK2 , PALB2, ATM, and NF1. Younger group: 6% (211/3493), genes: PALB2, CHEK2, TP53, ATM, PTEN, NF1, and NBN | (29) |
| Colombia | 2014 | Unselected women with BC (Medellin, Colombia) | 244 | BRCA1 and BRCA2 (recurrent hispanic BRCA mutations, BRCA1: 50 and BRCA2: 46) | 1.2% (3/244) | BRCA1: 0.8% (2/244); BRCA2: 0.4% (1/244) | NA | (30) |
|  | 2017 | Colombian breast/ovarian cancer families (small-range mutations); families (LGRs); unselected BC cases (Colombian founder mutations in BRCA1/2) | 68; 221; 1022 | BRCA1 and BRCA2 | Families (small-range mutations): 10.3% (7/68). Families (LGRs): 0.9% (2/221). Unselected BC: 7% (71/1022) | Families (small-range mutations): BRCA2: 8.8% (6/68), BRCA1: 1.5% (1/68). Families (LGRs): BRCA2: 0.9% (2/221). Unselected BC: BRCA1: 5.5% (56/1022); BRCA2: 1.5% (15/1022) | NA | (31) |
|  | 2018 | Women with HBOC fulfilling NCCN criteria | 85 | 25-gene hereditary cancer panel | 22.4% (19/85) | BRCA2: 9.4% (8/85); BRCA1: 8.2% (7/85) | 4.7% (4/85), genes: PALB2, ATM, MSH2, and PMS2 | (32) |
|  | 2019 | High-risk BC and/or OC families (Pacific region) | 58 | BRCA1 and BRCA2 | 20.7% (12/58) | BRCA2: 13.8% (8/58); BRCA1: 6.9% (4/58) | NA | (33) |
|  | 2022 | Afro-colombian families afected by BC/OC | 60 (BC: 51, BC and OC: 9) | BRCA1 and BRCA2 | BC: 3.9% (2/51); BC and OC: 33.3% (3/9) | BC: BRCA1: 3.9% (2/51). OC: BRCA1: 22.2% (2/9); BRCA2: 11.1% (1/9) | NA | (34) |
| Guatemala | 2021 | Unselected BC cases | 664 | BRCA1, BRCA2, PALB2, PTEN, TP53, ATM, BARD1, BRIP1, CHEK2, MSH6, RAD51D, and STK11 | 11% (73/664) | BRCA1: 5.6% (37/664), BRCA2: 2.3% (15/664) | 3.2% (21/664), genes: PALB2, TP53, ATM, CHEK2, BARD1, and MSH6 | (35) |
| Hispanic/Hispanic from Southern California | 2019 | Women with hereditary BC BRCA1/2 negative | 1054 | ATM, BRIP1, CDH1, CHEK2, NBN, NF1, PALB2, PTEN, RAD51C, RAD51D, STK11, and TP53 | 4.5% (47/1054) | NA | 4.5% (47/1054), genes: CHEK2, PALB2, ATM, BRIP1, TP53, CDH1, and NF1 | (36) |
| Latin America | 2021 | Participants (95.2% with cancer) from different countries of Latin America. Individuals were enrolled from Latin American CCGCRN | Total: 1627. Brazil (Porto Alegre, n=74), Colombia (Bogotá, n=225), Peru (Lima, n=653), Puerto Rico (San Juan, n=43), and Mexico (Guadalajara, n=94; Mexico City, n=538). | BRCA1 and BRCA2 (HISPANEL, semiconductor sequencing, and CNV detection) | 14.5% (236/1627); Brazil: 25.7% (19/74); Mexico: 17.4% (110/632); Peru: 12.6% (82/653); Colombia: 9.3% (21/225); Puerto Rico: 9.3% (4/43) | BRCA1: 9.8% (160/1627); BRCA2: 4.7% (76/1627) | NA | (37) |
| Latin american and US Hispanic | 2022 | Unrelated individuals with a personal/family history suggestive of HBOC. Group A (Mexico, Central America, and the Caribbean), group B (South America), and group C (United States with individuals who self-reported Hispanic ethnicity) | 24075 (331; 5867; 17877) | BRCA1, BRCA2, CHEK2, ATM, PALB2, and TP53, among others | Group A: 18.7% (62/331); Group B: 14% (814/5867); Group C: 9.1% (1626/17877) | Group A: BRCA1 7.2% (24/331); BRCA2: 6.9% (23/331). Group B: BRCA1: 4.3% (254/5867); BRCA2: 3.8% (224/5867). Group C: BRCA1: 2.7% (488/17877); BRCA2: 2.5% (443/17877) | Group A: 3.9% (13/331), genes: PALB2, ATM, MLH1, TP53, and MSH2. Group B: 4.2% (245/5867), genes: CHEK2, ATM, PALB2, TP53, and RAD51C. Group C: 2.3% (408/17877), genes: PALB2, ATM, CHEK2, RAD51C, and BRIP1 | (38) |
| Latin American | 2019 | Individuals who fulfilled NCCN criteria for HBOC from 11 health institutions of Latin America | 222. Argentina (n=57), Colombia (n=78), Guatemala (n=19), and Mexico (n=68). | 143 genes (Argentina, Guatemala and Mexico), 94 genes-TruSight Kit (Colombia). Patients from Colombia BRCA negative were tested for LGRs in BRCA1/2 genes through MLPA | 17% (38/222). Argentina: 17.5% (10/57); Guatemala: 15.8% (3/19); Colombia: 16.6% (13/78); Mexico: 17.6% (12/68) | Argentina: BRCA2: 10.5% (6/57); BRCA1: 3.5% (2/57). Guatemala: BRCA1: 10.5% (2/19). Colombia: BRCA2: 5.1% (4/78); BRCA1: 2.5% (2/78). Mexico: BRCA2: 5.9% (4/68); BRCA1: 5.9% (4/68) | Argentina: 3.5% (2/57), genes: WRN and MUTYH). Guatemala: 5.2% (1/19), gene: PDE11A. Colombia: 10.2% (8/78), genes: MSH2, MSH6, PDGFB, FANCM, DCLRE1C, PALB2, and MUTYH. Mexico: 5.9% (4/68), genes: ATM, MLH3, PALB2, and CDKN2A | (39) |
| Mexico | 2015 | 92 patients with ovarian cancer and 96 with breast cancer, both cohorts unselected for family history of cancer. | 188 | BRCA1 and BRCA2 | 21.3% (40/188); OC: 28% (26/92); BC: 15% (14/96) | BRCA1: 18.1% (34/188); BRCA2: 3.2% (6/188). OC: BRCA1: 25% (23/92); BRCA2: 3.3% (3/92). BC: BRCA1 11.4% (11/96), BRCA2: 3.1% (3/96) | NA | (40) |
|  | 2018 | Mexican patients with suspicion of HBOC and high-risk patients without cancer but with a severe family history of cancer | 300; 27 | 143 genes | HBOC: 15.3% (46/300); high-risk: 22% (6/27) | HBOC: BRCA1: 5% (15/300); BRCA2: 2% (6/300). High-risk: BRCA1: 7.4% (2/27); BRCA2: 3.7% (1/27) | HBOC: 8.3% (25/300), genes: ATM, PTEN, CHEK2,NBN, FANCI, ERCC3, ATR, FANCB, FANCC, FANCF, FANCL, FANCM, MLH1, RAD51C, POLH, RECQL4, SDHB and WRN. High-risk: 11% (3/27), genes: FANCF, PDE11A, and POLH | (41) |
|  | 2019 | Unrealted BC patients | 252 | BRCA1 and BRCA2 | 15.1% (38/252) | BRCA1: 10% (25/252); BRCA2: 5.1% (13/252) | NA | (42) |
|  | 2019 | Women with BC diagnosed at 45 years or younger and BRCA negative. Controls: mexican women aged 45 years or older without personal or family history of BC | 78; 509 | TP53 | 6.4% (5/78) | NA | 6.4% (5/78) | (43) |
|  | 2022 | Mexican patients with TNBC from CCGCRN | 387 | ATM, BRCA1, BRCA2, CDH1, CHEK2, NF1, PALB2, PTEN, RAD51C, RAD51D and TP53. MLPA for BRCA1 | 29.2% (113/387) | BRCA1: 23% (89/387); BRCA2: 4.4% (17/387) | 1.8% (7/387), genes: PALB2, ATM, BRIP1, PTEN, RAD51C, and TP53 | (44) |
|  | 2022 | Patients with breast cancer diagnosed at 40 years or younger | 115 | 862 cancer genes | 34% (39/115) | BRCA2: 4.3% (5/115), BRCA1: 1% (1/115) | 28.7% (33/115), genes: ATM, BLM, CHEK2, CLTCL1, COL3A1, CUX1, CYLD, DCC, DDX3X, ERCC1, ERCC6, EXT2, FANCE, FAT1, FLCN, FLG, C6PC3, HIF1A, MSH6, MUTYH, NFKBIE, NSD1, PALB2, PBRM1, PMS1, POLQ, PRDM9, PTCH2, RAD51C, RBM8A, RPS7, SPEN, TP53, TSC2, USP6, WRN, ZFHX3, and ZMYM3 | (45) |
| Peru | 2015 | Women with unselected BC | 266 | BRCA1 and BRCA2 (HISPANEL: 114 recurrent hispanic BRCA mutations) | 5% (13/266) | BRCA1: 4% (11/266); BRCA2: 1% (2/266) | NA | (46) |
|  | 2023 | BC and OC patients candidates for treatment with PARP inhibitors | 143; 382 | BRCA1 and BRCA2 (Ampliseq BRCA panel) | BC: 14.7% (21/143); OC: 20.7% (70/382) | BC: BRCA1: 9.1% (13/143), BRCA2: 5.6% (8/143). OC: BRCA1: 13.3% (51/382); BRCA2: 7.3% (28/382) | NA | (47) |
| Puerto Rico | 2019 | Women with BC diagnosis who fulfilled NCCN criteria and were BRCA1/2 negative | 48 | BROCA Agilent cancer risk panel (53 genes) | 8.3% (4/48) | NA | 8.3% (4/48), genes: CHEK2, MUTYH, and RAD51B | (48) |
| Spain and Peru | 2016 | Women with TNBC unselected by family history of breast or ovarian cancer (seven hospitals from Spain and one hospital located at Lima-Peru). | 105 | BRCA1, BRCA2, PALB2, BARD1, RAD50, RAD51C and RAD51D | 17.1% (18/105) | BRCA1: 12.4% (13/105); BRCA2: 1.9% (2/105) | 2.8% (3/105), genes: BARD1 and RAD51D | (49) |
| US Hispanic and Guatemala | 2024 | Unselected hispanic breast cancer cases from Texas (US Hispanics) and Guatemala | 96; 137 | Truncating variants: ATM, BRCA1, BRCA2, PALB2, CHEK2, BARD1, PTEN, RAD51C, RAD51D, TP53, BRIP1,CDH1, FANCC, FANCM, MSH2, MSH6, MUTYH, PMS2, and RAD50. Pathogenic missense variants: TP53, BARD1, RAD51C, and RAD51D | US Hispanics: 11.4% (11/96). Guatemala: 7.3% (10/137) | US Hispanics: BRCA1 3.1% (3/96); BRCA2: 3.1% (3/96). Guatemala: BRCA2: 3.6%; (5/137);BRCA1: 3% (4/137) | US Hispanics: 5.2% (5/96), genes: CHEK2, ATM, BARD1. Guatemala: 0.7% (1/137), gene: TP53 | (50) |

HBOC: Hereditary Breast Ovary Cancer; LFL: Li-Fraumeni Like syndrome; HBCC: Hereditary Breast and Colon Cancer syndrome; BOC: Breast Ovary Cancer; BC: Breast Cancer; OC: Ovary Cancer; CCGCRN: Clinical Cancer Genomics Community Research Network; PARP: Poly (ADP-ribose) Polymerase.

References

1. Richards S, Aziz N, Bale S, Bick D, Das S, Gastier-Foster J, et al. Standards and guidelines for the interpretation of sequence variants: a joint consensus recommendation of the American College of Medical Genetics and Genomics and the Association for Molecular Pathology. Genetics in Medicine. mayo de 2015;17(5):405-24.

2. Jarvik GP, Browning BL. Consideration of Cosegregation in the Pathogenicity Classification of Genomic Variants. The American Journal of Human Genetics. junio de 2016;98(6):1077-81.

3. Abou Tayoun AN, Pesaran T, DiStefano MT, Oza A, Rehm HL, Biesecker LG, et al. Recommendations for interpreting the loss of function PVS1 ACMG/AMP variant criterion. Human Mutation. noviembre de 2018;39(11):1517-24.

4. Ghosh R, Harrison SM, Rehm HL, Plon SE, Biesecker LG. Updated recommendation for the benign stand‐alone ACMG/AMP criterion. Human Mutation. noviembre de 2018;39(11):1525-30.

5. Walsh MF, Ritter DI, Kesserwan C, Sonkin D, Chakravarty D, Chao E, et al. Integrating somatic variant data and biomarkers for germline variant classification in cancer predisposition genes. Human Mutation. noviembre de 2018;39(11):1542-52.

6. Harrison SM, Biesecker LG, Rehm HL. Overview of Specifications to the ACMG/AMP Variant Interpretation Guidelines. Current Protocols in Human Genetics [Internet]. septiembre de 2019;103(1). Disponible en: https://currentprotocols.onlinelibrary.wiley.com/doi/10.1002/cphg.93

7. Brnich SE, Abou Tayoun AN, Couch FJ, Cutting GR, Greenblatt MS, Heinen CD, et al. Recommendations for application of the functional evidence PS3/BS3 criterion using the ACMG/AMP sequence variant interpretation framework. Genome Medicine. diciembre de 2020;12(1):3-3.

8. Vargas‐Parra G, Valle J, Rofes P, Gausachs M, Stradella A, Moreno‐Cabrera JM, et al. Comprehensive analysis and ACMG‐based classification of CHEK2 variants in hereditary cancer patients. Human Mutation. diciembre de 2020;41(12):2128-42.

9. Spurdle AB, Healey S, Devereau A, Hogervorst FBL, Monteiro ANA, Nathanson KL, et al. ENIGMA-Evidence-based network for the interpretation of germline mutant alleles: An international initiative to evaluate risk and clinical significance associated with sequence variation in BRCA1 and BRCA2 genes. Human Mutation. enero de 2012;33(1):2-7.

10. Solano AR, Liria NC, Jalil FS, Faggionato DM, Mele PG, Mampel A, et al. BRCA1 and BRCA2 Mutations Other Than the Founder Alleles Among Ashkenazi Jewish in the Population of Argentina. Front Oncol [Internet]. 21 de agosto de 2018 [citado 6 de mayo de 2024];8. Disponible en: https://www.frontiersin.org/journals/oncology/articles/10.3389/fonc.2018.00323/full

11. Cerretini R, Mercado G, Morganstein J, Schiaffi J, Reynoso M, Montoya D, et al. Germline pathogenic variants in BRCA1, BRCA2, PALB2 and RAD51C in breast cancer women from Argentina. Breast Cancer Research and Treatment. diciembre de 2019;178(3):629-36.

12. Solano AR, Mele PG, Jalil FS, Liria NC, Podesta EJ, Gutiérrez LG. Study of the Genetic Variants in BRCA1/2 and Non-BRCA Genes in a Population-Based Cohort of 2155 Breast/Ovary Cancer Patients, Including 443 Triple-Negative Breast Cancer Patients, in Argentina. Cancers (Basel). 31 de mayo de 2021;13(11):2711.

13. Gonzalez A, Del Greco F, Vargas-Roig L, Brun B, Tabares G, Mampel A, et al. PALB2 germline mutations in a multi-gene panel testing cohort of 1905 breast-ovarian cancer patients in Argentina. Breast Cancer Res Treat. julio de 2022;194(2):403-12.

14. Silva FC, Lisboa BC, Figueiredo MC, Torrezan GT, Santos ÉM, Krepischi AC, et al. Hereditary breast and ovarian cancer: assessment of point mutations and copy number variations in Brazilian patients. BMC Medical Genetics. 15 de mayo de 2014;15(1):55.

15. Palmero EI, Alemar B, Schüler-Faccini L, Hainaut P, Moreira-Filho CA, Ewald IP, et al. Screening for germline BRCA1, BRCA2, TP53 and CHEK2 mutations in families at-risk for hereditary breast cancer identified in a population-based study from Southern Brazil. Genet Mol Biol. 24 de mayo de 2016;39(2):210-22.

16. de Souza Timoteo AR, Gonçalves AÉMM, Sales LAP, Albuquerque BM, de Souza JES, de Moura PCP, et al. A portrait of germline mutation in Brazilian at-risk for hereditary breast cancer. Breast Cancer Res Treat. diciembre de 2018;172(3):637-46.

17. da Costa e Silva Carvalho S, Cury NM, Brotto DB, de Araujo LF, Rosa RCA, Texeira LA, et al. Germline variants in DNA repair genes associated with hereditary breast and ovarian cancer syndrome: analysis of a 21 gene panel in the Brazilian population. BMC Medical Genomics. 10 de febrero de 2020;13(1):21.

18. Sandoval RL, Leite ACR, Barbalho DM, Assad DX, Barroso R, Polidorio N, et al. Germline molecular data in hereditary breast cancer in Brazil: Lessons from a large single-center analysis. PLOS ONE. 19 de febrero de 2021;16(2):e0247363.

19. Gomes R, Spinola P da S, Brant AC, Matta BP, Nascimento CM, de Aquino Paes SM, et al. Prevalence of germline variants in consensus moderate-to-high-risk predisposition genes to hereditary breast and ovarian cancer in BRCA1/2-negative Brazilian patients. Breast Cancer Res Treat. febrero de 2021;185(3):851-61.

20. Felix GES, Guindalini RSC, Zheng Y, Walsh T, Sveen E, Lopes TMM, et al. Mutational spectrum of breast cancer susceptibility genes among women ascertained in a cancer risk clinic in Northeast Brazil. Breast Cancer Res Treat. junio de 2022;193(2):485-94.

21. Paixão D, Torrezan GT, Santiago KM, Formiga MN, Ahuno ST, Dias-Neto E, et al. Characterization of genetic predisposition to molecular subtypes of breast cancer in Brazilian patients. Frontiers in Oncology [Internet]. agosto de 2022;12. Disponible en: https://www.frontiersin.org/articles/10.3389/fonc.2022.976959/full

22. Guindalini RSC, Viana DV, Kitajima JPFW, Rocha VM, López RVM, Zheng Y, et al. Detection of germline variants in Brazilian breast cancer patients using multigene panel testing. Sci Rep. 9 de marzo de 2022;12(1):4190.

23. González-Hormazábal P, Castro VG, Blanco R, Gómez F, Peralta O, Waugh E, et al. Absence of CHEK2 1100delC mutation in familial breast cancer cases from a South American population. Breast Cancer Res Treat. 1 de agosto de 2008;110(3):543-5.

24. Gonzalez-Hormazabal P, Gutierrez-Enriquez S, Gaete D, Reyes JM, Peralta O, Waugh E, et al. Spectrum of BRCA1/2 point mutations and genomic rearrangements in high-risk breast/ovarian cancer Chilean families. Breast Cancer Res Treat. 1 de abril de 2011;126(3):705-16.

25. Gonzalez-Hormazabal P, Reyes JM, Blanco R, Bravo T, Carrera I, Peralta O, et al. The BARD1 Cys557Ser variant and risk of familial breast cancer in a South-American population. Mol Biol Rep. 1 de agosto de 2012;39(8):8091-8.

26. Leyton Y, Gonzalez-Hormazabal P, Blanco R, Bravo T, Fernandez-Ramires R, Morales S, et al. Association of PALB2 sequence variants with the risk of familial and early-onset breast cancer in a South-American population. BMC Cancer. 31 de enero de 2015;15(1):30.

27. Adaniel C, Salinas F, Donaire JM, Bravo ME, Peralta O, Paredes H, et al. Non-BRCA1/2 Variants Detected in a High-Risk Chilean Cohort With a History of Breast and/or Ovarian Cancer. J Glob Oncol. mayo de 2019;5:1-14.

28. Martin FJ, Saffie IM, Hurtado MA, Avila-Jaque D, Lagos RA, Selman CA, et al. Variants in BRCA1/2 in a hospital-based cohort in Chile and national literature review. Ecancermedicalscience. 2024;18:1683.

29. Chavarri-Guerra Y, Hendricks CB, Brown S, Marcum C, Hander M, Segota ZE, et al. The Burden of Breast Cancer Predisposition Variants Across The Age Spectrum Among 10 000 Patients. J Am Geriatr Soc. mayo de 2019;67(5):884-8.

30. Hernández JEL, Llacuachaqui M, Palacio GV, Figueroa JD, Madrid J, Lema M, et al. Prevalence of BRCA1 and BRCA2 mutations in unselected breast cancer patients from Medellín, Colombia. Hereditary Cancer in Clinical Practice. diciembre de 2014;12(1):11-11.

31. Torres D, Bermejo JL, Rashid MU, Briceño I, Gil F, Beltran A, et al. Prevalence and Penetrance of BRCA1 and BRCA2 Germline Mutations in Colombian Breast Cancer Patients. Scientific Reports. julio de 2017;7(1):4713-4713.

32. Cock-Rada AM, Ossa CA, Garcia HI, Gomez LR. A multi-gene panel study in hereditary breast and ovarian cancer in Colombia. Familial Cancer. enero de 2018;17(1):23-30.

33. Cifuentes-C L, Rivera-Herrera AL, Barreto G. BRCA1 and BRCA2 mutations in breast and ovarian cancer families from south west Colombia. Colombia Medica. 30 de septiembre de 2019;50(3):163-75.

34. Vargas E, de Deugd R, Villegas VE, Gil F, Mora L, Viaña LF, et al. Prevalence of BRCA1 and BRCA2 Germline Mutations in Patients of African Descent with Early-Onset and Familial Colombian Breast Cancer. The Oncologist. marzo de 2022;27(2):e151-7.

35. Ren M, Orozco A, Shao K, Albanez A, Ortiz J, Cao B, et al. Germline variants in hereditary breast cancer genes are associated with early age at diagnosis and family history in Guatemalan breast cancer. Breast Cancer Research and Treatment. septiembre de 2021;189(2):533-9.

36. Weitzel JN, Neuhausen SL, Adamson A, Tao S, Ricker C, Maoz A, et al. Pathogenic and likely pathogenic variants in PALB2, CHEK2, and other known breast cancer susceptibility genes among 1054 BRCA-negative Hispanics with breast cancer. Cancer. 15 de agosto de 2019;125(16):2829-36.

37. Herzog JS, Chavarri-Guerra Y, Castillo D, Abugattas J, Villarreal-Garza C, Sand S, et al. Genetic epidemiology of BRCA1- and BRCA2-associated cancer across Latin America. npj Breast Cancer. 19 de agosto de 2021;7(1):1-8.

38. Ossa Gomez CA, Achatz MI, Hurtado M, Sanabria-Salas MC, Sullcahuaman Y, Chávarri-Guerra Y, et al. Germline Pathogenic Variant Prevalence Among Latin American and US Hispanic Individuals Undergoing Testing for Hereditary Breast and Ovarian Cancer: A Cross-Sectional Study. JCO Global Oncology [Internet]. julio de 2022;(8). Disponible en: https://ascopubs.org/doi/10.1200/GO.22.00104

39. Oliver J, Quezada Urban R, Franco Cortés CA, Díaz Velásquez CE, Montealegre Paez AL, Pacheco-Orozco RA, et al. Latin American Study of Hereditary Breast and Ovarian Cancer LACAM: A Genomic Epidemiology Approach. Front Oncol. 2019;9:1429.

40. Villarreal-Garza C, Alvarez-Gómez RM, Pérez-Plasencia C, Herrera LA, Herzog J, Castillo D, et al. Significant clinical impact of recurrent BRCA1 and BRCA2 mutations in Mexico. Cancer. febrero de 2015;121(3):372-8.

41. Quezada Urban R, Díaz Velásquez CE, Gitler R, Rojo Castillo MP, Sirota Toporek M, Figueroa Morales A, et al. Comprehensive Analysis of Germline Variants in Mexican Patients with Hereditary Breast and Ovarian Cancer Susceptibility. Cancers. octubre de 2018;10(10):361.

42. Millan Catalan O, Campos-Parra AD, Vázquez-Romo R, Cantú de León D, Jacobo-Herrera N, Morales-González F, et al. A Multi-Center Study of BRCA1 and BRCA2 Germline Mutations in Mexican-Mestizo Breast Cancer Families Reveals Mutations Unreported in Latin American Population. Cancers. septiembre de 2019;11(9):1246.

43. Gallardo-Alvarado LN, Tusié-Luna MT, Tussié-Luna MI, Díaz-Chávez J, Segura YX, Bargallo-Rocha E, et al. Prevalence of germline mutations in the TP53 gene in patients with early-onset breast cancer in the Mexican population. BMC Cancer. 1 de febrero de 2019;19(1):118.

44. Chavarri-Guerra Y, Villarreal-Garza C, Ferrigno AS, Mohar A, Aguilar D, Alvarez-Gomez RM, et al. Germline pathogenic variants in Mexican patients with hereditary triple-negative breast cancer. Salud Publica Mex. 25 de febrero de 2022;64(1):41-8.

45. Gómez-Flores-Ramos L, Barraza-Arellano AL, Mohar A, Trujillo-Martínez M, Grimaldo L, Ortiz-Lopez R, et al. Germline Variants in Cancer Genes from Young Breast Cancer Mexican Patients. Cancers (Basel). 24 de marzo de 2022;14(7):1647.

46. Abugattas J, Llacuachaqui M, Allende YS, Velásquez AA, Velarde R, Cotrina J, et al. Prevalence of BRCA1 and BRCA2 mutations in unselected breast cancer patients from Peru. Clin Genet. octubre de 2015;88(4):371-5.

47. Ferreyra Y, Rosas G, Cock-Rada AM, Araujo J, Bravo L, Doimi F, et al. Landscape of germline BRCA1/BRCA2 variants in breast and ovarian cancer in Peru. Front Oncol [Internet]. 17 de agosto de 2023 [citado 6 de mayo de 2024];13. Disponible en: https://www.frontiersin.org/journals/oncology/articles/10.3389/fonc.2023.1227864/full

48. Dutil J, Teer JK, Golubeva V, Yoder S, Tong WL, Arroyo N, et al. Germline variants in cancer genes in high-risk non-BRCA patients from Puerto Rico. Sci Rep. 28 de noviembre de 2019;9(1):17769.

49. González-Rivera M, Lobo M, López-Tarruella S, Jerez Y, Del Monte-Millán M, Massarrah T, et al. Frequency of germline DNA genetic findings in an unselected prospective cohort of triple-negative breast cancer patients participating in a platinum-based neoadjuvant chemotherapy trial. Breast Cancer Res Treat. abril de 2016;156(3):507-15.

50. Godinez Paredes JM, Rodriguez I, Ren M, Orozco A, Ortiz J, Albanez A, et al. Germline pathogenic variants associated with triple-negative breast cancer in US Hispanic and Guatemalan women using hospital and community-based recruitment strategies. Breast Cancer Res Treat [Internet]. 23 de marzo de 2024 [citado 6 de mayo de 2024]; Disponible en: https://doi.org/10.1007/s10549-024-07300-2
